# Integrative transcriptomic-physiological analysis deciphers nitrogen-mediated carbon reallocation balancing growth and flavonoid metabolism in *Epimedium pubescens*

**DOI:** 10.3389/fpls.2025.1539445

**Published:** 2025-05-08

**Authors:** Shangnian Liu, Xiaojing An, Chaoqun Xu, Dongmei He, Xianen Li, Caixia Chen, Baolin Guo, De Xu, Juan Huang

**Affiliations:** ^1^ Key Laboratory of Bioactive Substances and Resources Utilization of Chinese Herbal Medicines, Ministry of Education & National Engineering Laboratory for Breeding of Endangered Medicinal Materials, Institute of Medicinal Plant Development, Peking Union Medical College and Chinese Academy of Medical Sciences, Beijing, China; ^2^ School of Pharmacy, State Key Laboratory of Characteristic Chinese Medicine Resources in Southwest China, Chengdu University of Traditional Chinese Medicine, Chengdu, China; ^3^ School of Chinese Materia Medica, Tianjin University of Traditional Chinese Medicine, Tianjin, China; ^4^ Institute of Chinese Materia Medica, Dazhou Academy of Agricultural Sciences, Dazhou, China

**Keywords:** nitrogen, *Epimedium pubescens*, flavonoid, icariin, carbon-nitrogen metabolism

## Abstract

Nitrogen availability critically shapes medicinal plant quality by coordinating the “growth–secondary metabolism” trade-off, yet its regulatory mechanisms remain elusive in the non-model species *Epimedium pubescens*. Through physiological-transcriptomic integration under five nitrogen levels (0, 3.5, 7.5,15, 22.5 mM NO_3_^−^), we demonstrated that moderate nitrogen (MN: 7.5 mM NO_3_^−^) optimally balanced biomass accumulation (22%–53% higher than low nitrogen [LN: 0 mM NO_3_^−^] and high nitrogen [HN: 22.5 mM NO_3_^−^]) with maximal Icariin-type flavonoid production (19%–34% higher than LN/HN). Extreme nitrogen stresses (LN/HN) impaired photosynthetic efficiency (18%–20% reduction), disrupted carbon–nitrogen homeostasis, and restricted flavonoid biosynthesis by hindering carbon reallocation (soluble sugars reduced by 26%–27%, starch by 30%–43%). Time-series transcriptomics revealed distinct response dynamics: LN triggered active transcriptional reprogramming at mid-stage (36 days after treatment, DAT), whereas HN responses were delayed to late-stage (48 DAT). Weighted gene co-expression network analysis (WGCNA) identified the grey60 module as a hub coordinating carbon–nitrogen metabolism and mRNA processing. A tripartite regulatory network linking nitrogen-responsive genes (e.g., *EpF3H*, *UGT*), Icariin-type flavonoid/carbon metabolism (e.g., icariin, soluble sugars), and growth phenotypes (e.g., biomass, photosynthesis) elucidated how nitrogen optimizes the trade-off between medicinal quality and yield in *E. pubescens*. This study provides molecular targets for precision nitrogen management to enhance both medicinal quality and yield, while establishing an integrative framework combining physiological and transcriptomic analyses to investigate metabolic trade-offs in non-model plants.

## 1 Introduction

Nitrogen serves as the most critical macronutrient for plant growth and development, playing a fundamental role in synthesizing essential biomolecules including proteins, nucleic acids, and amino acids, while also participating in organic metabolites production and plant signaling pathways (Laursen, 2020). As a primary limiting factor for agricultural productivity, improper nitrogen availability significantly impairs carbon metabolism in plants (Hongbiao et al., 2008), ultimately compromising crop yield and quality (Hadj et al., 2010; Yao et al., 2019; Mu, 2021).

The photosynthetic machinery, being the initial stage of plant carbon metabolism, exhibits particular sensitivity to nitrogen availability (Hohmann-Marriott and Blankenship, 2011; Bracher et al., 2017). Nitrogen deficiency induces chlorophyll degradation and protein breakdown in foliar tissues (Havé et al., 2017; Wen et al., 2022), thereby disrupting photosynthesis efficiency. In *Panax notoginseng.* Both high and low nitrogen levels concentrations suppress carbon metabolism through distinct mechanisms: elevated nitrogen downregulates carbon assimilation genes and reduces Rubisco activity, while nitrogen limitation decreases expression of Calvin cycle enzymes and light-harvesting complex proteins (Zhang et al., 2020).

However, to prevent yield decline due to nitrogen (N) deficiency, excessive nitrogen fertilization has become prevalent in agricultural practice. Excessive nitrogen application can inhibit root elongation and reduce nitrogen use efficiency (NUE) in plants (Wang et al., 2010), disrupting their carbon-nitrogen metabolic balance and consequently impairing both yield and quality. For instance, rice cultivation frequently demonstrates compromised grain quality and reduced productivity under excessive nitrogen application (Dechorgnat et al., 2011; Gutierrez, 2012). Furthermore, excessive nitrogen fertilizer can lead to an increase in pests and diseases (Chen et al., 2008), destabilize soil ecosystems, and exacerbate production costs while contributing to environmental pollution through nitrate leaching and nitrous oxide emissions (Yin et al., 2007; Shaobing et al., 2010). Consequently, elucidating plant responses to differential nitrogen availability, developing N-efficient cultivars, and optimizing nitrogen management strategies for enhanced utilization efficiency constitute critical research priorities in sustainable crop production.

*Epimedium pubescens* Maxim. (*E. pubescens*), a perennial herb in the genus *Epimedium* in the Berberidaceae family, is valued for its foliage utilized as the traditional Chinese medicine (TCM) “Yin Yang Huo”. It possesses the efficacies of nourishing kidney yang, strengthening bones and muscles, and dispelling rheumatism and dampness (Ma et al., 2011), maintaining an irreplaceable status in TCM formulations. The leaf-derived icariin-type flavonoids (prenylated flavonol glycosides from *Epimedium* spp.) exhibit diverse pharmacological activities such as immunomodulation, sexual function enhancement, antioxidant properties, and cardiovascular protection (Wu et al., 2003; Ma et al., 2011), thus demonstrating promising application potential in modern TCM and healthcare product development. Current natural populations of *E. pubescens* remain limited and insufficient to meet growing market demand. Consequently, artificial cultivation has become essential for resolving supply-demand disparities and ensuring sustainable resource utilization. Within cultivation systems, nitrogen fertilization serves as a critical management practice for optimizing both biomass production and secondary metabolite accumulation.

Flavonoids, as a predominant class of plant secondary metabolites, playing a pivotal role in plant adaptation to biotic and abiotic stresses, including nutrient deprivation, thermal fluctuations, and environmental perturbations (Lillo et al., 2008; Kumar and Pandey, 2013; Li et al., 2023). Nitrogen availability profoundly modulates flavonoid biosynthesis by regulating the allocation of carbon skeletons and energy substrates between primary and secondary metabolic pathways. Elevated nitrogen levels in tea plants (cv. Longjing43) enhance free amino acids and organic acids in roots and mature leaves, concomitant with a marked reduction in soluble sugar and flavonoid content in young shoots, indicative of carbon flux redirection towards nitrogen assimilation at the expense of phenylpropanoid metabolism (Liu et al., 2021b). Similarly, this trade-off is corroborated in Capsicum annuum, where increased nitrogen application inversely correlates with fruit flavonoid content (Zhang et al., 2024). In *Epimedium pseudowushanense* (*E. pseudowushanense*), optimal nitrogen supply(7–13 mM) maximizes biomass production and icariin flavonoid accumulation, whereas nitrogen limitation severely suppresses photosynthetic capacity (chlorophyll content, Pn) and flavonoid synthesis (Zhang et al., 2017). Nevertheless, the mechanistic interplay between nitrogen levels, carbon-nitrogen metabolic crosstalk, and icariin-type flavonoid accumulation in *E. pubescens* remains elusive. Based on previous research reports, we hypothesize that under nitrogen deficiency and excess stress conditions, nitrogen availability governs carbon partitioning in *E. pubescens* through metabolic reprogramming, thereby coordinating plant growth-defense trade-offs and icariin-type flavonoid biosynthesis.

To test this hypothesis, we quantified growth parameters, primary carbon metabolites (soluble sugars, starch), nitrogen status (total N), and icariin flavonoid compounds in *E. pubescens* leaves under differential nitrogen regimes. Integrating genomic resources (Shen et al., 2022) and developmental transcriptome data of icariin flavonoid biosynthesis (Xu et al., 2023), we performed comparative transcriptomics to delineate nitrogen-responsive regulatory networks. Through weighted gene co-expression network analysis (WGCNA), we constructed a stress-responsive core gene regulatory framework orchestrating carbon-nitrogen metabolic crosstalk and icariin-type flavonoid biosynthesis under nitrogen deficiency and excess stress conditions, identifying pivotal hub genes and thresholds for stress adaptation. These findings establish a theoretical foundation for precision nitrogen management in stress-resilient *E. pubescens* cultivation, development of nitrogen-deficiency/excess-tolerant genotypes, and targeted genetic enhancement of medicinal compound production under fluctuating nitrogen environments.

## 2 Materials and methods

### 2.1 Plant material and experimental design

In October 2022, one-year-old healthy homogeneous seedlings of *Epimedium pubescens* Maxim. (Berberidaceae) exhibiting comparable developmental stages (5–7 simple leaves and 1–3 trifoliate leaves) were acquired from commercial cultivation bases in Dazhou City, Sichuan Province (31°28′19.90″N, 107°39′8.37″E). Taxonomic authentication was performed by Prof. Baolin Guo (Institute of Medicinal Plant Development, Chinese Academy of Medical Sciences) with voucher specimens (B.L.Guo 0711-3) deposited in the Herbarium of the same institution (Liu et al., 2024).

Seedlings were transplanted into 25 cm × 30 cm cultivation pots containing a 3:2 (v/v) substrate mixture of river sand and horticultural perlite (3:2), then acclimatized in a controlled-environment greenhouse under 14/10 h photoperiod (2000 luX illumination), 22 ± 2°C ambient temperature, and 60-80% relative humidity. Following 20-day pre-cultivation with 12.5% Hoagland nutrient solution (1.82 mM NO_3_^-^; pH 6.5), seedlings demonstrating successful root regeneration and stable growth were selected for nitrogen treatments. Five nitrate concentration gradients were established: LN (Low Nitrogen, 0 mM NO_3_^-^), LM (Low to Medium Nitrogen, 3.75 mM NO_3_^-^), MN (Medium Nitrogen, 7.5 mM NO_3_^-^), MH (Medium to High Nitrogen, 15 mM NO_3_^-^) and HN (High Nitrogen, 22.5 mM NO_3_^-^). Nutrient solutions were formulated using KNO_3_ and Ca (NO_3_)_2_ as primary nitrogen sources, supplemented with NaNO_3_ for target nitrate adjustments. Ionic balance was maintained through compensatory additions of CaCl_2_ and K_2_SO_4_, with micronutrient concentrations standardized as: 2.86 μM H_3_BO_3_, 1.81 μM MnCl_2_, 0.22 μM ZnSO_4_, 0.08 μM CuSO_4_, 0.02 μM H_2_MoO_4_, and 0.02 mM Fe-EDTA (full composition in **Supplementary Table S1**). The pH of the nutrient solution was adjusted to 6.5. Each plant received 200 mL of nutrient solution per irrigation event, a volume determined to saturate the substrate and ensure complete root immersion, which was administered at 10-day intervals.

Each treatment involved 36 seedlings. Fresh leaves from plants under different treatments were collected at three developmental time points (24, 36, and 48 days after treatment initiation, DAT), rapidly frozen in liquid nitrogen, and then stored at -80°C to maintain RNA quality for transcriptome profiling.

At 48 DAT, plants in the LN (0 mM NO_3_^-^) and HN (22.5 mM NO_3_^-^) treatments showed obvious symptoms of damage: chlorosis (LN) in older leaves vs. necrosis (HN) in older leaves. Subsequently, all treated plants underwent final phenotyping followed by destructive sampling at this stage.

### 2.2 Photosynthetic properties

Photosynthetic parameters were measured using a LI-6400 Portable Photosynthesis System (LI-COR, USA) on mature leaves at the same leaf position (the second compound leaf counted from the rootstock apex) of plants between 8:00-11:00 AM at 48 DAT. Concurrently, SPAD values (relative chlorophyll content) of the same leaves were quantified with an SPAD-502 Chlorophyll Meter (Konica Minolta Inc., Japan) employing 650/940 nm dual-wavelength detection.

### 2.3 Sample collection

Leaf samples were collected at 24, 36, and 48 DAT from different treated plants were collected (3 replicates per treatment) and immediately frozen in liquid nitrogen, then stored at -80°C for future testing. By 48 DAT plants from the LN and HN treatments exhibited significant symptoms of damage. Subsequently, all treated plants underwent systematic processing: biometric measurement, organ separation (roots, rhizomes, stems, leaves), and desiccation at 45°C until constant weight (3 replicates per treatment). ​​The total dry weight of all leaves harvested from a single plant was used as the leaf dry weight for that plant sample, and the dry weights of roots, rhizomes, stems, and other parts were similarly determined.

### 2.4 Detection of nitrogen content

Weigh 0.5 g of dried and constant-weight *E. pubescens* leaf powder, transfer it into a digestion tube, digest it using sulfuric acid-hydrogen peroxide, and then determine the total nitrogen content by the Nash colorimetric method (Bremner, 1960).

### 2.5 Detection of soluble sugar and starch content

Dried leaf powder of *E. pubescens* (50.0 mg, constant weight) was weighed into a 10 mL centrifuge tube. 80% ethanol (4.0 mL) was added, and the mixture was sealed and incubated in an 80°C water bath for 30 minutes (duplicate extraction). After centrifugation at 3000 rpm for 5 minutes, the supernatant was collected, mixed with 10 mg activated charcoal, and decolorized at 80°C for 15 min. The solution was then diluted to 25 mL with distilled water, filtered, and used as the test solution for soluble sugar content.

The residue was air-dried and transferred toa 25 mL centrifuge tube. Distilled water (15mL) was added, followed by incubation in a boiling water bath for 15 min. Subsequently,2 mL of 9.2 mol/L perchloric acid solution was added, and the mixture was boiled for another 15 min. After cooling to room temperature, the volume was adjusted to 25 mL with distilled water to obtain the starch test solution.

For quantification, 0.2 mL of the test solution was mixed with 6 mL of anthrone reagent, vortexed thoroughly, and immediately heated at 95°C 15 min. The absorbance was measured at 620 nm after natural cooling. Soluble sugar and starch contents were calculated using a pre-established standard curve:

Calibration curve: (Y = 0.0017X + 0.0075, R^2^ = 0.9993), where X = glucose concentration (μg/mL), Y = absorbance, with a linear range of 40–500 μg/mL.

### 2.6 Detection of Icariin-type flavonoids content

The content of Icariin-type flavonoids in *E. pubescens* leaves was determined by UPLC method (Xu et al., 2023). Briefly, 100 mg of dried leaf powder was subjected to ultrasonic extraction with 10 mL of 50% ethanol for 30 minutes, and then filtered through a 0.22 μm filter membrane. Chromatographic analysis was performed using an ACQUITYTM Ultra-Performance Liquid Chromatography (UPLC) system (Waters Corporation, USA) equipped with an ACQUITY UPLC BEH C18 column (2.1 mm × 100 mm, 1.7 μm). The mobile phase consisted of solvent A (water) and solvent B (acetonitrile). The gradient elution program was set as follows: 0 min, 79% A; 6 min, 71% A; 12 min, 56% A; 17 min, 5% A. Detection was performed at a wavelength of 270 nm, under which the retention times of the Icariin-type flavonoids were observed as follows: Epimedin A: 5.45 min, Epimedin B: 5.75 min, Epimedin C: 6.10 min, and Icariin: 6.40 min (**Supplementary Figure S1**).

### 2.7 Transcriptome analysis

Total RNA extraction from plant leaves was performed using the RNAprep Pure Polysaccharide/Polyphenol Plant Total RNA Extraction Kit (QIAGEN, Germany). The quality and integrity of the RNA were assessed using the Agilent 2100 Bioanalyzer (Agilent Technologies, USA). All samples met RNA integrity criteria (RIN >7) with total RNA quantities ≥400 ng. For RNA sequencing (RNA-seq) library preparation, three biological replicates were sequenced for each sample using the Illumina NovaSeq 6000 sequencing platform (Illumina, USA). Gene expression levels were normalized using Fragments Per Kilobase Million (FPKM) to control for sequencing depth and transcript length.

HISAT2 v2.0.5 was employed to align the sequencing data to the reference genome (*E. pubescens* (Shen et al., 2022), and Stringtie 1.3.3b (Pertea et al., 2015) was used to identify known gene transcripts and predict novel gene transcripts. Differential gene expression between treatments was compared using the DESeq2 software (1.20.0). The resulting P-values were adjusted using the Benjamini & Hochberg method to control the false discovery rate, and genes with a *p*-adj < 0.05 and |log_2_FC| ≥ 1 were considered as differentially expressed genes (DEGs). The clusterProfiler (3.8.1) software was employed to conduct GO and KEGG enrichment analysis on the DEGs.

### 2.8 Real-time quantitative PCR validation

The extracted RNA was reverse transcribed into cDNA using an M-MLV Reverse Transcriptase Kit (Zoman Biotech, Beijing, China) following this protocol: 1 μg total RNA was mixed with 5 μL 4×RT Mix and ddH_2_O to a final volume of 20 μL. After gentle vertexing, the reaction was incubated at 45°C for 15 min followed by 85°C for 5 min to inactivate the enzyme. The synthesized cDNA was diluted 10-fold for subsequent analysis. Ten candidate genes involved in flavonoid biosynthesis, selected from S3-stage differential genes, were validated, with *β-Actin-1* serving as the internal reference gene. The details of the selected genes and their primer sequences are provided in **Supplementary Table S2**.

Quantitative PCR was performed using 2×HQ SYBR qPCR Mix (Without Rox; Zoman Biotech) with the following reaction system: 1 μL diluted cDNA, 0.4 μL forward primer (10 μM), 0.4 μL reverse primer (10 μM), and 10 μL qPCR Mix in a total volume of 20 μL. Three technical replicates per gene and three biological replicates per treatment were analyzed. Each gene was subjected to three technical replicates, and each treatment sample was replicated biologically three times. Gene expression was quantified via the 2^−ΔΔCt^ method to validate transcriptome reliability. Complete thermal cycling conditions are described in **Supplementary Table S3**.

### 2.9 Statistical analysis

Each experiment was conducted using a completely randomized design with three replicates, and the data were analyzed using the statistical software SPSS 26.0. One-Way ANOVA was employed to analyze the differences among different treatments at a significance level of *p* < 0.05.

## 3 Result

### 3.1 Plant growth and development

**Figure 1:**
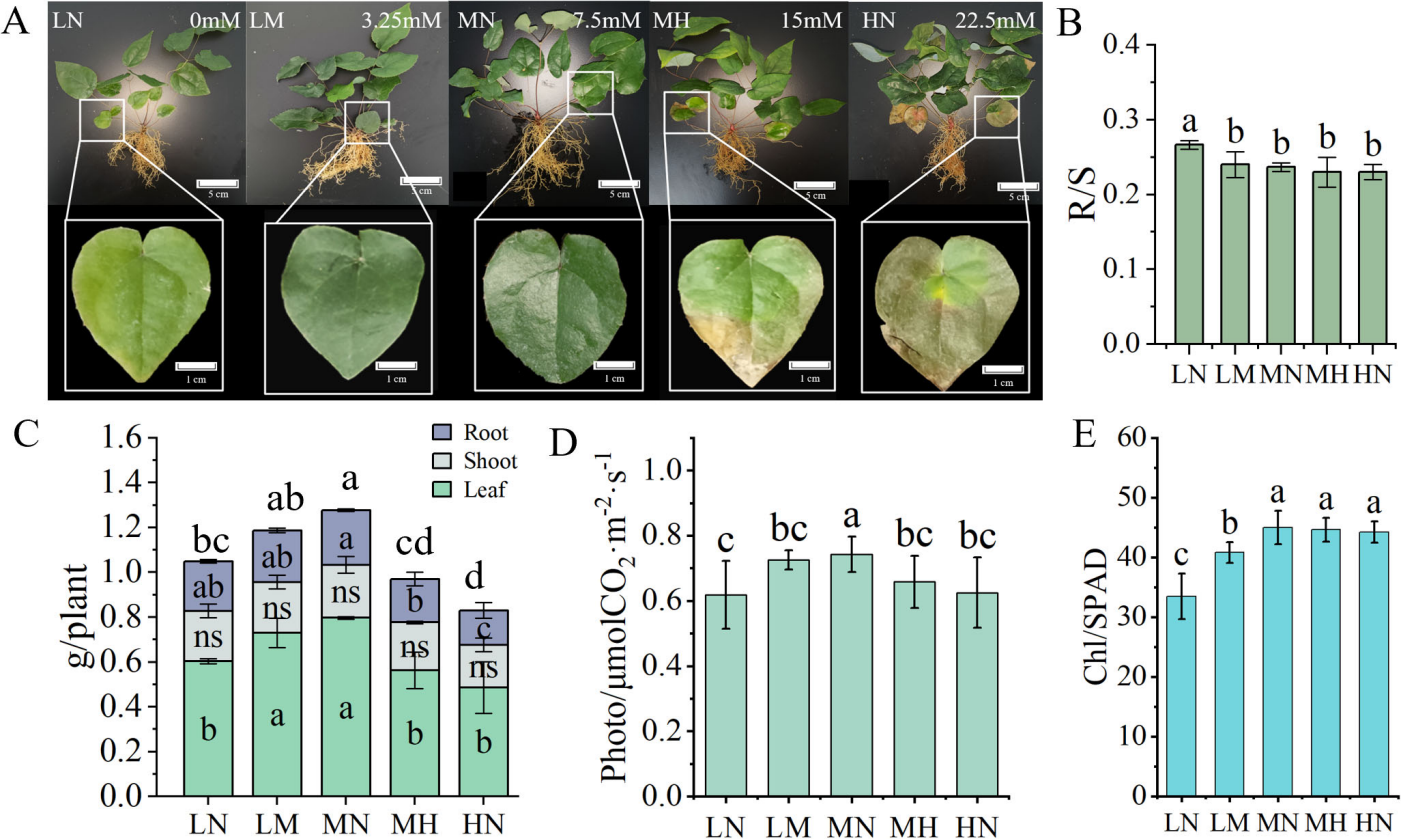
Differences in growth appearance and indicators of *E. pubescens* among different nitrogen treatment levels at the S3 stage (48 DAT). **(A)** Morphological characteristics and performance parameters of older leaves at the plant base in *E*. *pubescens* were assessed at the S3 stage (48 DAT) under varying nitrogen levels; **(B, C)** Root-to-shoot (R/S) ratio(B), and dry weight (g) of plant different parts (leaves, stems, roots), and total plant biomass (g) **(C)** of *E*. *pubescens* under different nitrogen treatment levels; **(D, E)** Photosynthetic indices including photosynthetic rate (μmol CO_2_·m^-2^·s^-1^) **(D)** and relative chlorophyll content (SPAD) **(E)**. Different lowercase letters indicate significant differences between treatments; the letters above the bars in **(C)** represent differences in total plant biomass (One-Way ANOVA, n = 3, *p* < 0.05).

Growth indicators of *E. pubescens* varied under different nitrogen treatment levels (**Figure 1**). Older leaves at the plant base displayed distinct nitrogen-related visual changes: LN-treated plants developed pale green coloration (chlorosis), whereas MH/HN treatments triggered leaf midrib yellowing and edge-to-base browning. Notably, plants under MN treatment (7.5 mM NO_3_^−^) retained normal dark green leaves without visible stress symptoms, suggesting ideal nitrogen supply (**Figure 1A**).

Organ-specific biomass partitioning in *E. pubescens* was quantified under different nitrogen level treatments, with whole-plant measurements encompassing leaves, roots, and stems (**Figure 1C**). Whole-plant leaf dry weight (LDW) varied significantly among nitrogen treatments: LN (24% reduction, 0 mM NO_3_^−^), MH (29% reduction, 15 mM NO_3_^−^) and HN (39% reduction, 22.5 mM NO_3_^−^) treatments exhibited significantly lower leaf dry weight compared to the MN treatment (*p* < 0.05). Root biomass exhibited particular sensitivity to excess nitrogen, with HN plants producing 30% less root mass than MN (*p* < 0.05). Among the treatment levels, there were no significant differences in stem dry weight. However, significant variations in total plant dry weight were observed, with the MN treatment exhibiting the highest total plant dry weight (increase 22% vs. LN, increase 54% vs. HN, *p* < 0.05), followed by the LN treatment, and the HN treatment showing the lowest. These results indicate that moderate nitrogen levels (MN) optimize biomass accumulation in *E. pubescens*, while both nitrogen deficiency (LN) and excess (MH/HN) reduce total plant productivity, with the strongest suppression observed in leaf biomass.

To gain a deeper understanding of how varying nitrogen levels impact the aboveground-to-belowground biomass allocation in *E. pubescens*, we calculated and compared the R/S ratios (root-to-shoot ratio, calculated as belowground biomass divided by aboveground biomass) across different treatment levels (**Figure 1B**). Notably, the R/S ratio in the LN treatment was significantly higher than that in the other treatment levels, suggesting that under nitrogen deficiency, the plants allocated a greater proportion of carbon to their root systems. This enhanced root growth led to increased biomass accumulation, ultimately elevating the root-to-shoot ratio (indicating higher belowground investment). This phenomenon could be interpreted as an adaptive response of *E. pubescens* to nitrogen deficiency stress, whereby increased root growth facilitates nitrogen uptake. Conversely, excessive nitrogen stress (HN) significantly inhibited root growth (30% reduction vs. MN, *p* < 0.05, **Figure 1C**). These findings further elucidate the growth performance of *E. pubescens* in adapting to different nitrogen levels, emphasizing that an optimal nitrogen level (MN) is conducive to the growth and rational biomass allocation of *E. pubescens*.

Different nitrogen levels significantly affected the photosynthetic characteristics of *E. pubescens* leaves (**Figures 1D, E**). The photosynthetic rate under MN (0.74 ± 0.05 μmol CO_2_·m^-2^·s^-1^) was significantly higher than other levels (20% increase vs. LN, 18% increase vs. HN, *p* < 0.05, **Figure 1D**). Chlorophyll content peaked under MN, showing no significant difference from MH and HN but was markedly higher than LN and LM (**Figure 1E**). The photosynthetic rate under MN was significantly higher than other treatment levels. Chlorophyll content peaked under MN, showing no significant difference from the MH and HN, but was markedly higher than in the LN and LM. The LN treatment exhibits the lowest chlorophyll content, which is significantly lower than that in the other treatment levels. These results indicates that moderate nitrogen addition promotes plant photosynthesis and chlorophyll synthesis, whereas excessive nitrogen (beyond a threshold) inhibits photosynthesis and reduces chlorophyll synthesis efficiency. This trend align with plant biomass distribution patterns, confirming that that photosynthetic capacity is closely linked to plant biomass accumulation. Thus, optimizing nitrogen levels is critical for enhancing photosynthetic performance and biomass production.

### 3.2 Nitrogen content, carbon metabolites and icariin-type flavonoids in leaves

**Figure 2:**
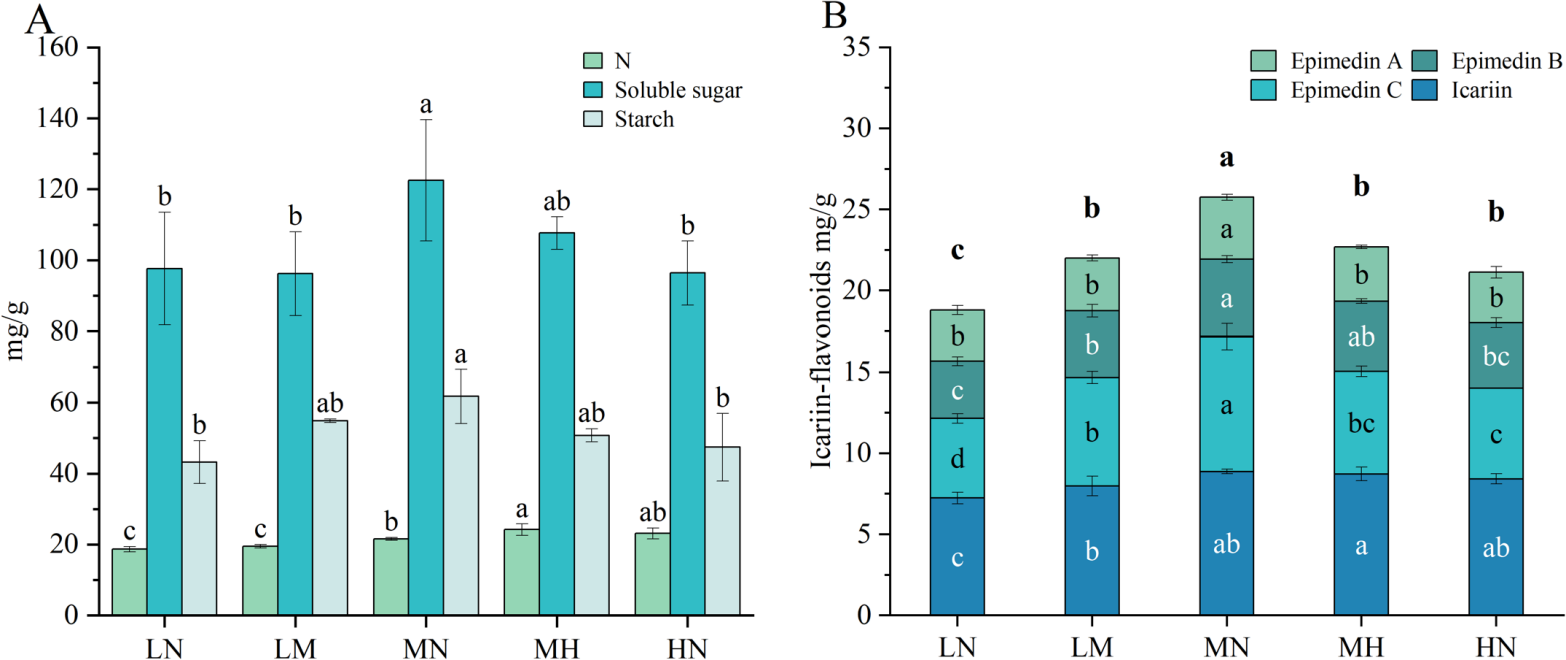
Metabolite and nitrogen concentrations in the leaves of *E*. *pubescens* at the S3 stage (48 DAT) were assessed under different nitrogen levels of treatment. **(A)** Soluble sugar, starch, and nitrogen content (mg/g) in the leaves of *E*. *pubescens* under different nitrogen levels; **(B)** The content of Icariin-type flavonoids in the leaves of *E. pubescens* under different nitrogen levels of treatment. Different lowercase letters represent significant differences between treatments (n = 3, *p* < 0.05); the letters above the bars in **(B)** represent differences in total flavonoid content (Sum of the contents of Epimedin A, Epimedin B, Epimedin C, and Icariin).

There exists a close interaction between nitrogen metabolism and carbon metabolism in plants, which synergistically regulate the growth, development, and metabolism of plants. To further understand the effects of different nitrogen levels on the carbon and nitrogen metabolism of plants, this study measured the nitrogen content and main carbohydrate components in leaves (**Figure 2A**). The results for nitrogen content indicate that the LN and LM have the lowest nitrogen levels in their leaves, significantly lower than that of the MN. In contrast, the MH and HN exhibit significantly higher nitrogen content in their leaves compared to the MN, but there is no significant difference existed between the MH and HN. In fact, the nitrogen content in the leaves of the HN treatment shows a slight decline. These findings indicate that nitrogen deficiency reduces the nitrogen content in leaves, while nitrogen application significantly increases it; however, excessive nitrogen application yielded diminishing returns on nitrogen accumulation.

Calculations and comparisons of the nitrogen use efficiency (NUE) of leaves, using the formula NUE_bala_ = nitrogen uptake after application/total nitrogen applied (Quan et al., 2021). The results showed that NUE_LM_ was 1.64 times that of NUE_MN_, 4.17 times that of NUE_MH_, and 7.58 times that of NUE_HN_, NUE_MN_ was 4.62 times that of NUE_HN_ (NUE for LN was excluded due to zero nitrogen input). Our findings clearly demonstrate that NUE decreases with increasing nitrogen application rates, with the most pronounced decline observed in the high nitrogen treatments (MH and HN). This indicates that excessive nitrogen supply markedly reduces nitrogen use efficiency in *E. pubescens*.

In terms of carbon metabolism, soluble sugars and starch, as key products of this process, show content changes closely linked to photosynthesis efficiency. Both components peaked under MN (soluble sugars: 122.54 ± 17.11 mg/g, starch: 61.77 ± 7.62 mg/g), significantly exceeding levels in other treatments (soluble sugars: 26% increase vs. LN, 27% increase vs. HN; starch: 43% increase vs. LN, 30% increase vs. HN, *p* < 0.05, **Figure 2A**). This finding underscores the profound impact of nitrogen supply on plant carbon metabolism. Moderate nitrogen stimulates carbon metabolism, enhancing accumulation of key products like soluble sugars and starch, whereas nitrogen deficiency or excess disrupts these processes, impairing plant growth and productivity.

The content of Icariin-type flavonoids (main active component) in *E. pubescens* leaves varied significantly across nitrogen levels. Total flavonoids and individual compounds (Epimedin A, Epimedin B, Epimedin C, Icariin) and in *E. pubescens* leaves peaked under MN treatment. Except for Icariin and Epimedin B, whose contents showed no significant difference between MN and HN treatments, all components in MN were significantly higher than other treatments. Conversely, LN had the lowest content of all compounds, with Icariin, Epimedin B, and Total flavonoids significantly reduced compared to other levels (**Figure 2B**). These results indicate that optimal nitrogen levels promotes Icariin-type flavonoid accumulation in leave (Total flavonoids: 24.98 ± 0.80 mg/g, 34% increase vs. LN, 19% increase vs. HN, *p* < 0.05, **Figure 2B**), while nitrogen deficiency and excess significantly inhibit this process, with deficiency showing stronger suppression. This underscores the crucial role of appropriate nitrogen supply in Icariin-type flavonoid synthesis in *E. pubescens* leaves.

### 3.3 Differential expressed genes and functional enrichment in leaves

The appearance, biomass, and metabolite content of plants showed obvious differences in different nitrogen levels. LN (nitrogen deficiency), MN (nitrogen suitability), and HN treatment (nitrogen excess) were selected as three representatives of different nitrogen levels, and transcriptome analysis was carried out to reveal the internal mechanism of different nitrogen levels regulating plant growth and metabolism. Transcriptome sequencing results of the LN, MN, and HN treatments during the S1-S3 stages (24–48 DAT) ultimately yielded a total of 1,244,447,328 clean reads, representing 186.67G of clean data. The average Q20 quality score was 97.99%, the average Q30 quality score was 94.37%, and the average GC content was 44.92%. Alignment of the clean data from each sample with the *E. pubescens* reference genome achieved a robust alignment rate ranging from 84.01% to 89.11%.

**Figure 3:**
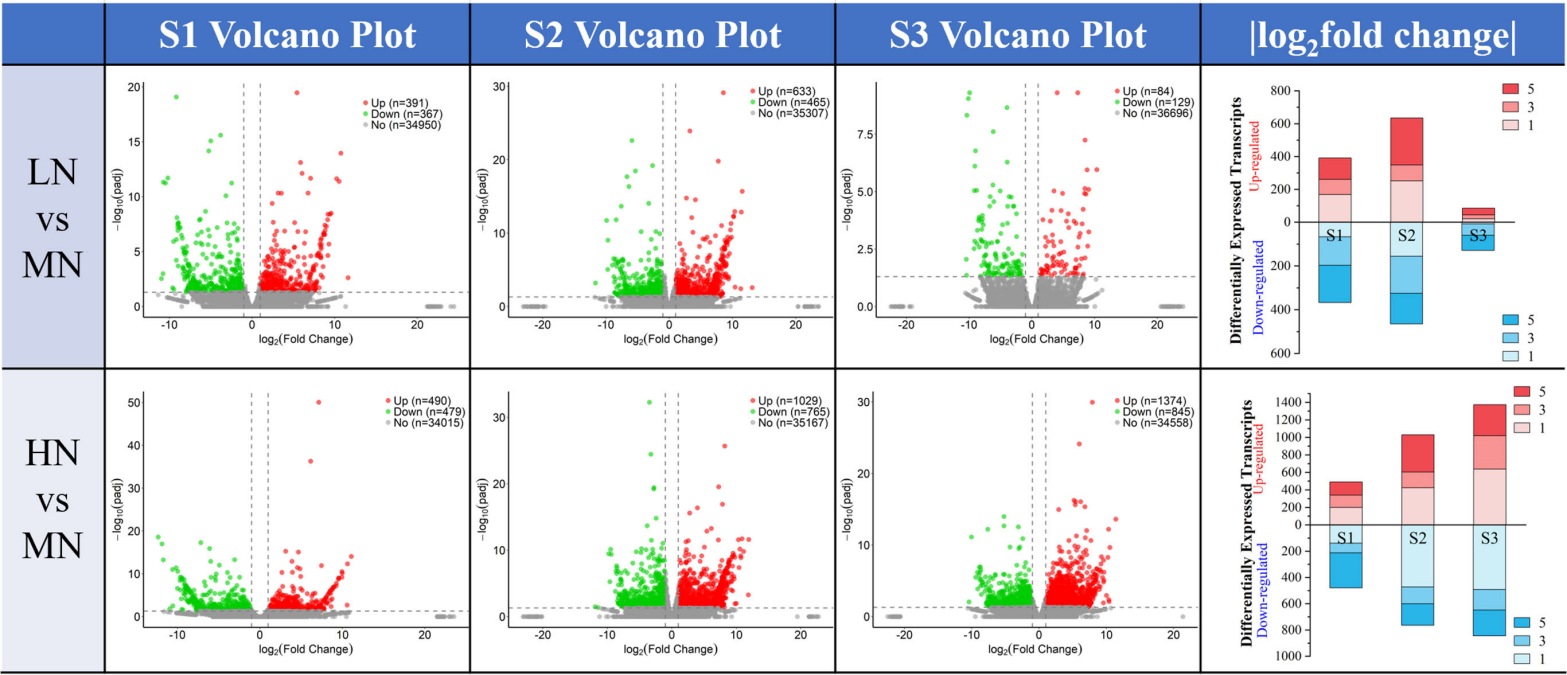
The number of DEGs in leaves between LN vs MN and HN vs MN at three stages (S1-S3, 24–48 DAT). ​​The volcano plot illustrates the number of upregulated (red) and downregulated (blue) differentially expressed genes (DEGs) across different time periods (S1 to S3, 24 to 48 DAT) under varying treatment comparisons, along with their fold change (|log_2_FC|, x-axis) and significance (-log_10_ *p*-adj, y-axis); The bar chart integrates the changes in the number of upregulated (red) and downregulated (blue) DEGs (y-axis) and their fold differences across the three periods (categorized on the y-axis), allowing clear visualization of DEG variations over time. The legend indicating the |log_2_FC| values.

Comparisons were made among the transcriptome sequencing results of leaves at S1, S2, and S3 stages under different treatments. Compared with the MN treatment, the LN treatment showed 391 upregulated differentially expressed genes (DEGs) and 367 downregulated DEGs at the S1 stage; 633 upregulated DEGs and 465 downregulated DEGs at the S2 stage; and a significant reduction in the number of DEGs at the S3 stage, with more downregulated DEGs observed, specifically 84 upregulated and 129 downregulated DEGs. In contrast, the HN treatment had 490 upregulated DEGs and 479 downregulated DEGs at the S1 stage; 1029 upregulated and 765 downregulated DEGs at the S2 stage; and a notable increase in the number of DEGs at the S3 stage, with 1374 upregulated and 845 downregulated DEGs (**Figure 3**).

**Figure 4:**
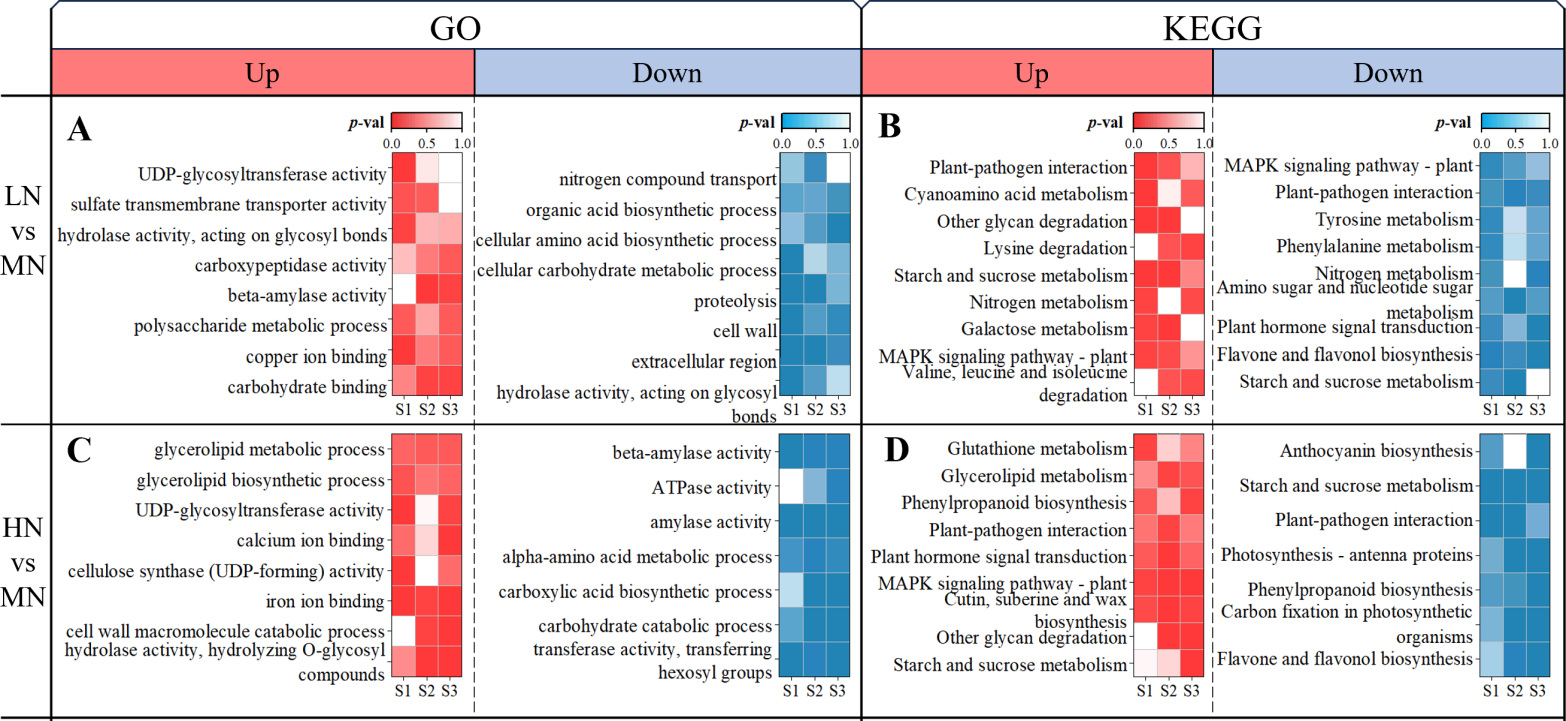
Stage-specific functional enrichment of DEGs under LN and HN treatments. **(A)** GO enrichment for LN vs MN comparisons; **(B)** KEGG enrichment for LN vs MN; **(C)** GO enrichment for HN vs MN; **(D)** KEGG enrichment for HN vs MN, analyzed at S1 (24 DAT), S2 (36 DAT), and S3 (48 DAT). Color intensity corresponds to the significance level (*p*-value).

For stages S1-S3 (24–48 DAT), Gene Ontology (GO) and Kyoto Encyclopedia of Genes and Genomes (KEGG) enrichment analyses were conducted on the up- and down-regulated DEGs obtained from LN and HN treatments, respectively, compared to the MN treatment, with results displayed in **Figure 4** (GO: left; KEGG: Right).

**GO Enrichment:** In the LN treatment, genes up-regulated relative to MN were significantly enriched in GO categories associated with carbohydrate binding and metabolism, copper ion binding, and sulfate transport (**Figure 4A**). Specifically, “UDP-glycosyltransferase activity”, “copper ion binding”, and “sulfate transmembrane transporter activity” exhibited the strongest enrichment in S1, whereas “carbohydrate binding”, “beta-amylase activity”, and “polysaccharide metabolic process” became prominent in S2 and S3. In contrast, genes down-regulated under LN were enriched in functional terms related to cell wall organization, carbon metabolism, and nitrogen processes. Among these, “cell wall”, “extracellular region”, and “organic acid biosynthetic process” showed the most marked suppression in S1, followed by reduced “proteolysis” and “nitrogen compound transport” in S2, and repression of “cellular amino acid biosynthetic process” in S3.

Under HN treatment, up-regulated genes were enriched in GO categories associated with carbohydrate hydrolysis, cell wall macromolecule catabolism, and ion binding (**Figure 4C**). Notably, “cellulose synthase (UDP-forming) activity” and “iron ion binding” dominated the enrichment profile in S1, while “hydrolase activity (hydrolyzing O-glycosyl compounds)”, “cell wall macromolecule catabolic process”, and “calcium ion binding” were most prominent in S3. Conversely, down-regulated genes in HN were linked to amino acid and carbon metabolism, with “transferase activity (transferring hexosyl groups)” and “amylase activity” showing the strongest suppression in S1, followed by repressed “carbohydrate catabolic process” and “carboxylic acid biosynthetic process” in S2, and sustained down-regulation of “alpha-amino acid metabolic process” in both S2 and S3.

**KEGG Enrichment:** Compared to MN, genes up-regulated under LN treatment were enriched in pathways including “Other glycan degradation”, “Cyanoamino acid metabolism”, “Galactose metabolism”, “Lysine degradation”, and “Valine, leucine, and isoleucine degradation” (**Figure 4B**). Among these, “Other glycan degradation” and “Cyanoamino acid metabolism” showed the strongest enrichment in S1, while “Galactose metabolism” peaked in S2. Conversely, down-regulated genes in LN were associated with pathways such as “Flavone and flavonol biosynthesis”, “Phenylalanine metabolism”, “Tyrosine metabolism”, and “Plant hormone signal transduction”, with the latter two exhibiting the most marked suppression in S3. Additionally, pathways like “Starch and sucrose metabolism” and “Nitrogen metabolism” were dynamically regulated, appearing in both up- and down-regulated DEGs across stages, with “Starch and sucrose metabolism” displaying the strongest up-regulation in S2.

For HN treatment, up-regulated genes were enriched in pathways such as “Other glycan degradation”, “Cutin, suberine, and wax biosynthesis”, “Plant hormone signal transduction”, and nitrogen-related pathways including “Tryptophan metabolism”, “Glycerolipid metabolism”, and “Glutathione metabolism” (**Figure 4D**). “Glutathione metabolism” dominated the enrichment profile in S1, followed by “Tryptophan metabolism” in S3, while other pathways peaked in S2.

Compared to MN, genes down-regulated under both LN and HN treatments were enriched in pathways associated with photosynthesis and carbon metabolism, including “Carbon fixation in photosynthetic organisms” and “Photosynthesis-antenna proteins” (**Figures 4B, D**). Similarly, phenylpropanoid and flavonoid synthesis pathways such as “Flavone and flavonol biosynthesis”, “Anthocyanin biosynthesis”, and “Phenylpropanoid biosynthesis” exhibited marked suppression, with the strongest down-regulation observed in S3 across both treatments (**Figures 4B, D**). The “Starch and sucrose metabolism” pathway displayed dynamic regulation, appearing in both up- and down-regulated DEGs. Up-regulation of this pathway peaked in S3 under LN treatment (**Figure 4B**), while down-regulation persisted from S1 to S3 under HN treatment, showing the strongest suppression in S1 (**Figure 4D**). Additionally, some common stress response pathways in plants, such as “Plant-pathogen interaction” and “MAPK signaling pathway-plant” (Takahashi et al., 2011; Zhu et al., 2023), were significantly enriched in the DEGs of LN and HN treatments, respectively.

**Figure 5:**
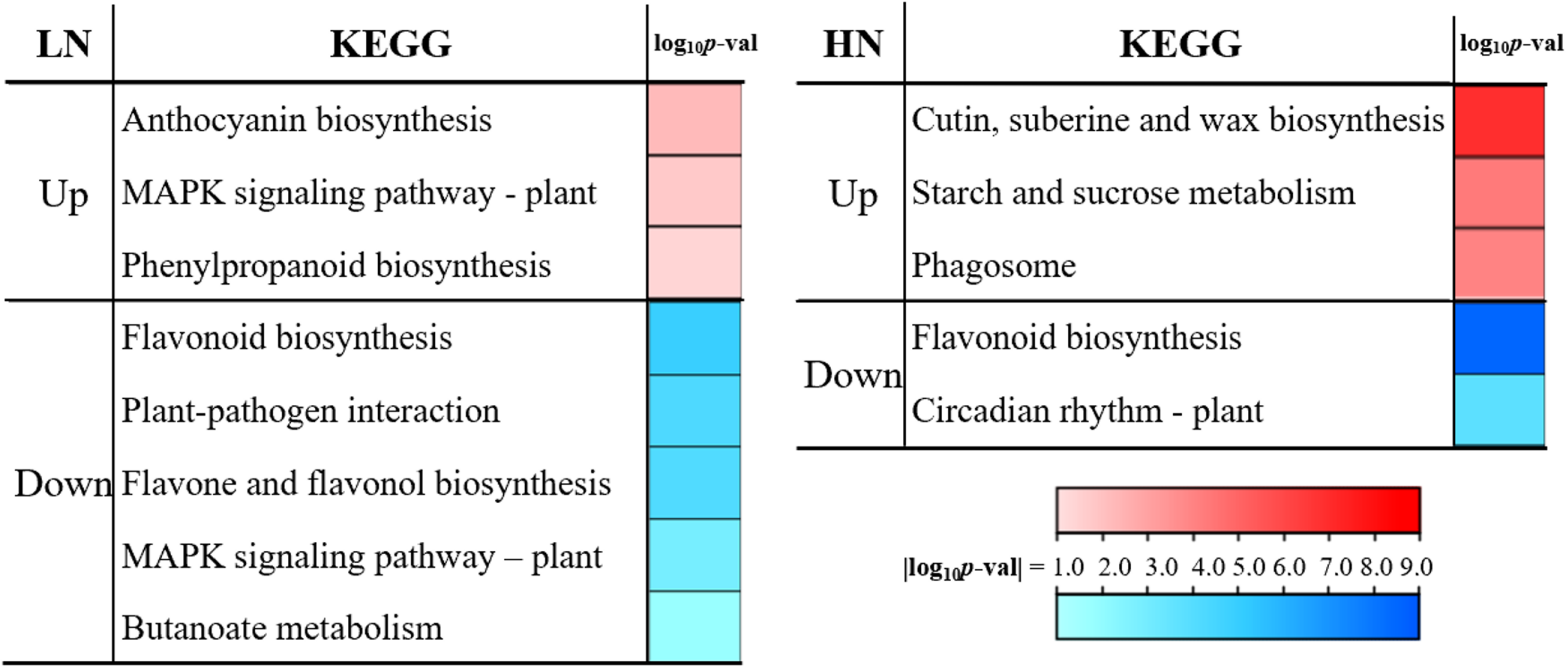
Stage transition (S1 to S3) KEGG pathway enrichment under LN and HN treatments. Significance is represented by color gradient (|log_10_ p-value|).

To investigate dynamic transcriptional responses of *E. pubescens* to nitrogen deficiency (LN) and excess (HN) over time, we analyzed stage-specific differentially expressed genes (DEGs) between S1 and S3 under both treatments, followed by KEGG enrichment (**Figure 5**).LN Treatment (S1 vs. S3): Genes up-regulated in S3 relative to S1 were enriched in pathways associated with stress adaptation, including “Anthocyanin biosynthesis”, “MAPK signaling pathway-plant”, and “Phenylpropanoid biosynthesis”. Conversely, down-regulated genes were linked to secondary metabolism and biotic interactions, with marked suppression of “Flavonoid biosynthesis”, “Plant-pathogen interaction”, “Flavone and flavonol biosynthesis”, and “Butanoate metabolism”.

HN Treatment (S1 vs. S3): Up-regulated genes in S3 were enriched in pathways related to structural biosynthesis and energy mobilization, notably “Cutin, suberine and wax biosynthesis”, “Starch and sucrose metabolism”, and “Phagosome”. In contrast, down-regulated genes showed reduced activity in “Flavonoid biosynthesis” and “Circadian rhythm-plant”, suggesting disrupted metabolic and physiological synchronization.

### 3.4 Weighted gene co-expression network analysis

**Figure 6:**
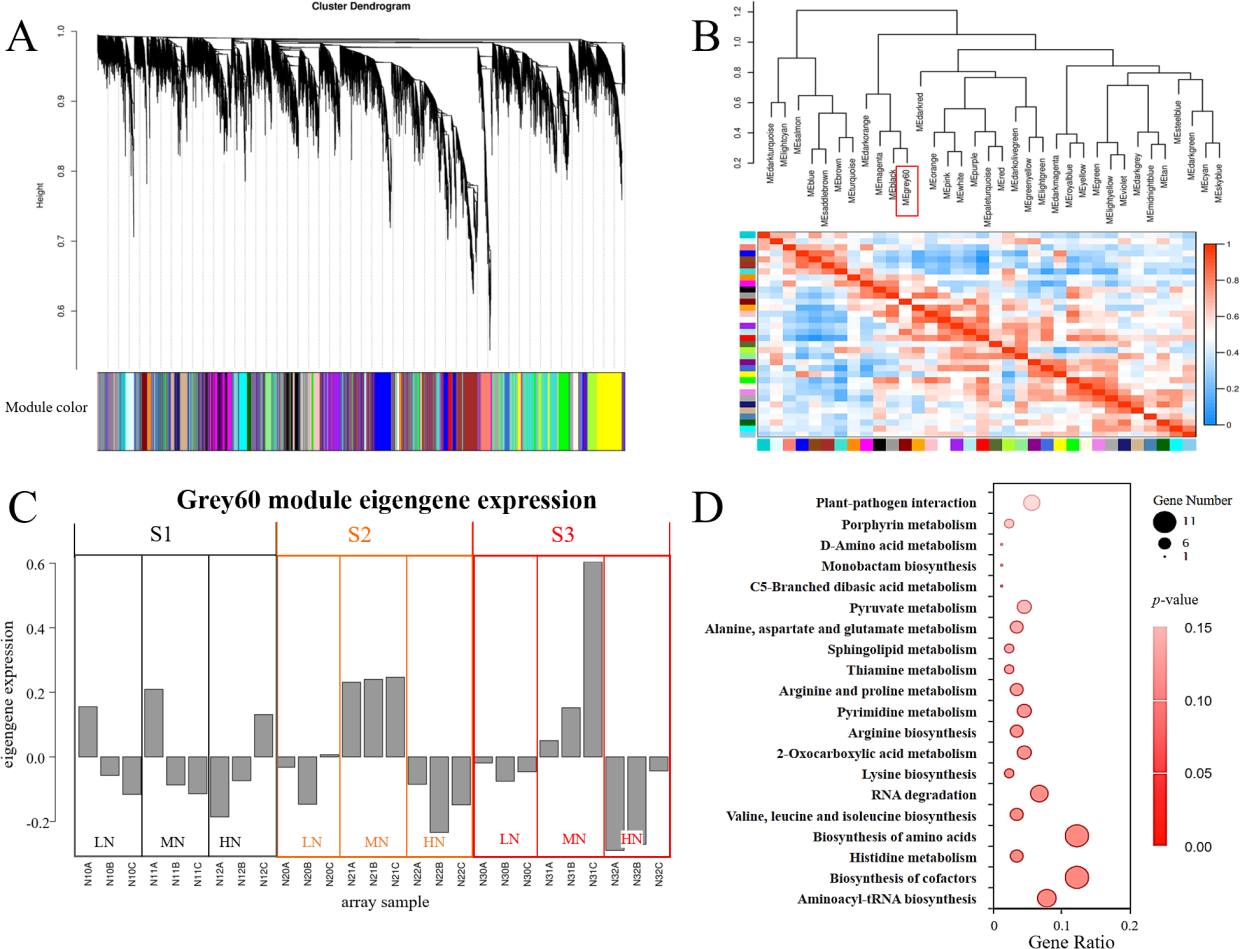
Weighted Gene Co-expression Network Analysis (WGCNA) of leaf transcriptomes in *E*. *pubescens* under nitrogen treatments. **(A)** Hierarchical clustering dendrogram of genes. Leaves (vertical lines) represent individual genes, with branches colored to indicate 35 distinct co-expression modules. **(B)** Module-trait correlation matrix. Cell colors (from blue to red) denote Pearson correlation coefficients between module eigengenes. **(C)** Expression patterns of the grey60 module across treatments (LN, MN, HN) and stages (S1-S3; 24–48 DAT). **(D)** KEGG pathways enriched in the grey60 module.

Weighted Gene Co-expression Network Analysis (WGCNA) is a systems biology approach for identifying highly correlated gene clusters (modules) across experimental conditions, widely applied in plant stress response studies (Langfelder and Horvath, 2008). To elucidate dynamic gene network reorganization in *E. pubescens* under nitrogen variation, we performed WGCNA on transcriptomes from S1-S3 stages of LN, MN, and HN treatments (**Figure 6A**). Modules were defined with a minimum eigengene correlation threshold of 0.75, yielding 35 distinct co-expression networks (**Figure 6B**). Among these, the grey60 module exhibited contrasting expression patterns between MN and nitrogen-stressed (LN/HN) treatments at S2-S3 (**Figure 6C**), suggesting its potential role in nitrogen adaptation.

KEGG enrichment of the grey60 module revealed marked involvement in three functional clusters: Translation fidelity and RNA regulation: Enriched pathways included “Aminoacyl-tRNA biosynthesis” and “RNA degradation”; Metabolic coordination: “Biosynthesis of cofactors”, “Porphyrin metabolism”, “Sphingolipid metabolism”, and amino acid biosynthesis pathways (“Valine, leucine and isoleucine biosynthesis”, “Biosynthesis of amino acids”, “Histidine metabolism”); Carbon-energy interface: “2-Oxocarboxylic acid metabolism” and “Pyruvate metabolism”. Additionally, “Plant-pathogen interaction” pathway genes were significantly enriched (**Figure 6D**), implying crosstalk between nitrogen stress and biotic defense mechanisms.

### 3.5 Changes in genes related to carbon metabolism, nitrogen metabolism, and Icariin-type flavonoid metabolism

**Figure 7:**
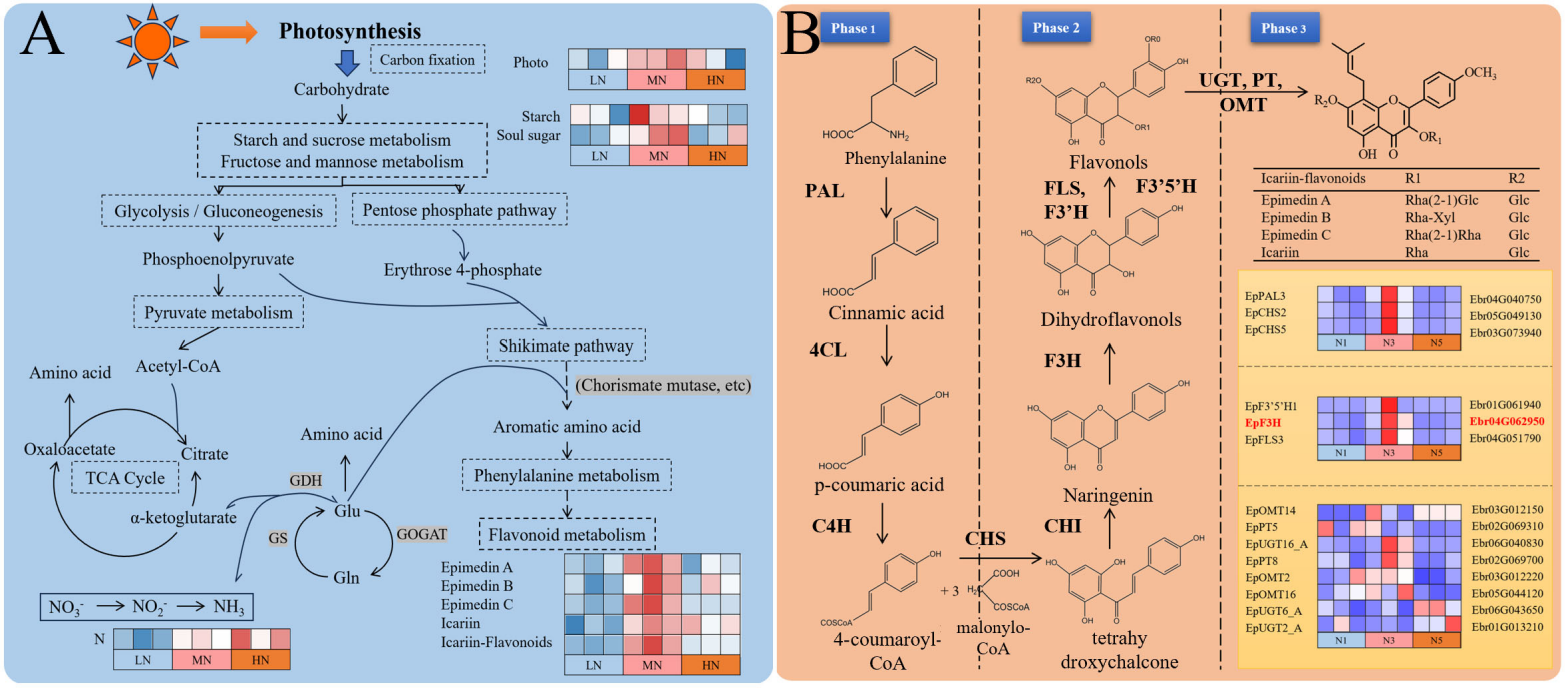
Integrated carbon-nitrogen-flavonoid metabolic network and transcriptional regulation in *E*. *pubescens*. **(A)** Schematic of metabolic crosstalk between carbon/nitrogen assimilation and flavonoid biosynthesis. The heatmap (right) shows relative levels of key metabolites (rows) and photosynthetic parameters (columns) across treatments (LN, MN, HN). **(B)** Heatmap of gene expression in the icariin-type flavonoid pathway. Rows: enzyme-coding genes (annotated by EC number); columns: treatments (LN, HN) and stages (S1-S3). Gene IDs are listed on the right.

Flavonoids, the primary medicinal components of *E. pubescens*, play dual roles in plant defense against biotic/abiotic stresses and nutrient deprivation (Lillo et al., 2008; Kumar and Pandey, 2013; Li et al., 2023). These phenylalanine-derived secondary metabolites are synthesized through a well-characterized pathway (Holton and Cornish, 1995; Koes et al., 2005), where carbon skeletons from primary metabolism enter the shikimate pathway, while nitrogen metabolism-derived amino acids contribute to phenylalanine production, ultimately fueling flavonoid biosynthesis (**Figure 7A**). Icariin-type flavonoid synthesis occurs in three phases: Phase 1: The enzymes phenylalanine ammonia-lyase (PAL), cinnamate 4-hydroxylase (C4H), and 4-coumarate-CoA ligase (4CL) sequentially convert phenylalanine to p-coumaroyl-CoA. Phase 2: Chalcone synthase (CHS) and chalcone isomerase (CHI) transform p-coumaroyl-CoA into chalcone, the precursor for flavonols/flavones (Dao et al., 2011). Subsequently, flavonoid 3’,5’-hydroxylase (F3’5’H), flavanone 3-hydroxylase (F3H), flavonoid 3’-hydroxylase (F3’H), and flavonol synthase (FLS) modify chalcone into diverse flavonols. Phase 3: UDP-glycosyltransferases (UGTs), prenyltransferases (PTs), and O-methyltransferases (OMTs) mediate glycosylation, prenylation, and O-methylation of flavonols, yielding icariin-type flavonoids (**Figure 7B**).

During the S3 stage, the number of differentially expressed genes (DEGs) in plants under nitrogen deficiency stress was significantly reduced (**Figure 3**), consistent with previous findings under phosphorus deficiency stress, indicating that nitrogen deficiency stress had entered the late stress phase by this stage. In contrast, the temporal dynamics of DEGs under excess nitrogen stress diverged from those under nitrogen or phosphorus deficiency. Specifically, excess nitrogen exhibited a sustained increase in DEG numbers, with an 85% rise from S1 to S3 (24 to 48 DAT) but only a 25% increase from S2 to S3 (36 to 48 DAT). The deceleration in the upward trend suggests that transcriptional responses had stabilized during this period. Consequently, the transcriptional strategy adopted by plants at this stage reflects a steady state following prolonged stress resistance, which may more accurately reflect plant adaptation mechanisms to distinct nitrogen stress conditions. Under inappropriate nitrogen (LN/HN) at S3 stage (48 DAT), transcriptional suppression was observed across all icariin-type flavonoid biosynthesis phases (**Figure 7B**): Phase 1: *PAL* expression was markedly reduced. Phase 2: Key genes (*CHS*, *F3H*, *FLS*, *F3’5’H*) showed significant downregulation. Phase 3: Most *PT* and *OMT* genes were suppressed, though partial *UGT* upregulation occurred. Notably, upregulated *UGT*/*OMT* isoforms may participate in alternative carbon metabolic routes rather than flavonoid synthesis, as substrate availability declined due to Phase 1–2 suppression and reduced photosynthetic input.

**Figure 8:**
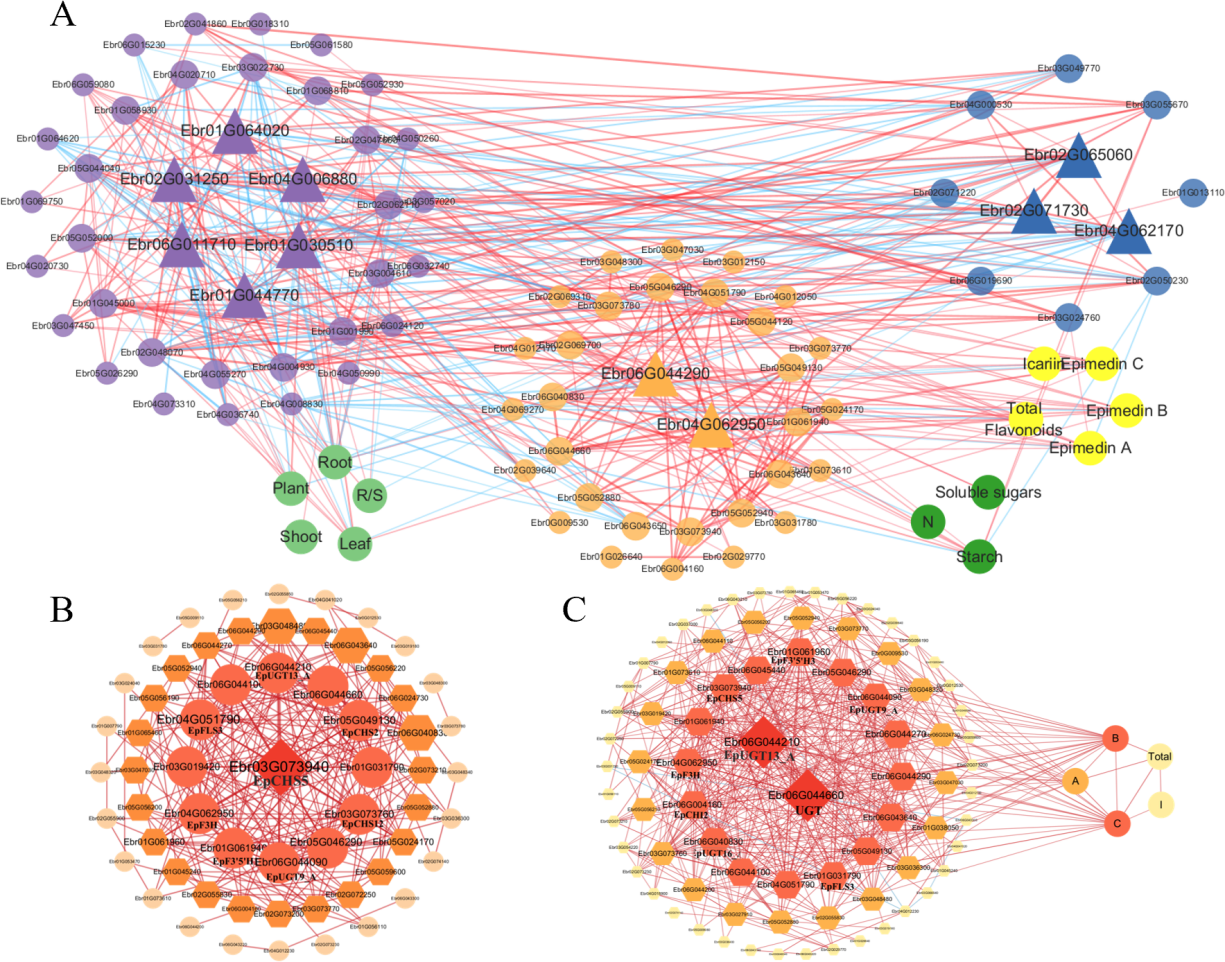
Integrated correlation networks of metabolic pathways and flavonoid biosynthesis in *E*. *pubescens*. **(A)** Tripartite correlation network integrates three categories: genes [carbon metabolism genes (purple nodes), nitrogen metabolism genes (blue nodes), and Epimedium icariin-type flavonoid biosynthetic genes (orange-yellow nodes)], metabolites [icariin-type flavonoids (bright yellow nodes), carbon metabolites (green nodes)], and physiological traits (light green nodes). **(B)** Correlation network diagram of genes related to Icariin-type flavonoid synthesis. **(C)** Network diagram combining genes related to Icariin-type flavonoid synthesis with the content of Icariin-type flavonoids. A/B/C, Epimedin A/B/C; I, Icariin. Edges represent Pearson correlations (|*r*| > 0.70): red = positive, blue = negative; thickness scales with |*r*|. Hub genes (triangular or prism nodes) were identified by maximal node degree.

To delineate metabolic crosstalk among carbon, nitrogen, and flavonoid pathways, we integrated S3-stage DEGs enriched in these pathways (**Figure 7**) to reconstruct a tripartite correlation network (**Supplementary Table S4**). For flavonoid metabolism refinement, we performed gene set enrichment analysis (GSEA) using localized software (http://www.broadinstitute.org/gsea) to identify additional candidate genes ( (Subramanian et al., 2005); **Supplementary Table S5**). Subsequent Pearson correlation analysis of 84 pathway-related genes, 5 biomass parameters, and 8 metabolites (|r| > 0.70 threshold) revealed interconnected regulatory networks, visualized through Cytoscape (**Figure 8**). The resultant network comprised 95 nodes with 467 edges, with hub genes identified by maximal node degree: Carbon metabolism: *Alpha-amylase* (Ebr01G064020), *Transcriptional activator* (Ebr06G011710), *Phosphoglucomutase* (Ebr01G030510), *Fructokinase-2* (Ebr02G031250), *Fructose-1,6-bisphosphatase* (Ebr01G044770). Nitrogen metabolism: *Amidase* (Ebr04G062170), *Aromatic aminotransferase* (Ebr02G065060), *Lamin-like protein* (Ebr02G071730). Flavonoid metabolism: *UGT* (UDP-glycosyltransferase; Ebr06G044290), *EpF3H* (*Ebr04G062950*).

Subnetworks focusing on flavonoid biosynthesis (**Figure 8B**) and gene-flavonoid associations (**Figure 8C**) highlighted additional regulators: *EpCHS5* (*Ebr03G073940*; chalcone synthase), *UGT* (*Ebr06G044660*), *EpUGT13_A* (*Ebr06G044210*).

Notably, Epimedin B/C exhibited stronger correlations with flavonoid pathway genes than other icariin-type flavonoids, suggesting their transcriptional responsiveness to metabolic perturbations.

### 3.6 *MYB1* and *MYB12* regulate flavonoid metabolism in response to nitrogen levels

**Figure 9:**
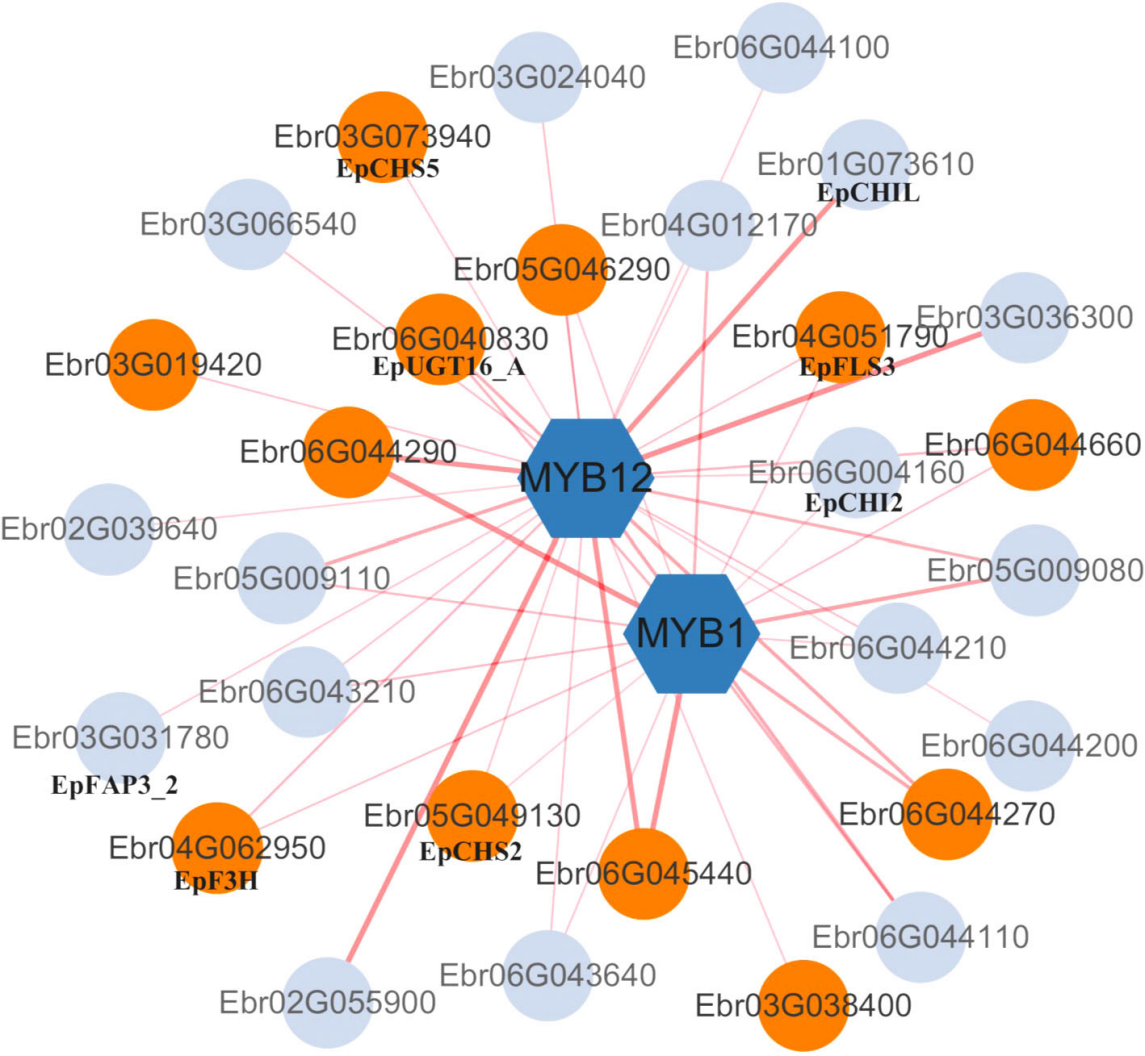
Co-expression network of MYB transcription factors and flavonoid metabolism-related genes. Orange-red nodes represent differentially expressed genes (DEGs; |log_2_FC| > 1, *p*-adj < 0.05). Edges indicate significant Pearson correlations (|r| > 0.70, *p* < 0.01), with thickness proportional to |r|.

Among DEGs in S3 stage (48DAT), 88 transcription factors were identified, including *MYB*, *NAC*, *WRKY* families. *NAC* and *WRKY* are known stress-responsive TFs in plants (Wani et al., 2021), while the *MYB* family is typically involved in regulating flavonoid biosynthesis pathways (Liu et al., 2015; Naik et al., 2022). Co-expression analysis revealed that *MYB1* (Ebr04G001770) and *MYB12* (Ebr01G065030) showed strong correlations (Pearson >0.9) with *UGT* genes (Ebr06G045440, Ebr06G044290). Notably, *MYB12* exhibited broader network connectivity than *MYB1* (**Figure 8**), suggesting its potential prominence in nitrogen-responsive flavonoid regulation. Key flavonoid genes (e.g., *EpFLS3*, *EpF3H*) displayed varying correlation strengths with both TFs across N levels (**Figure 9**). These patterns align with known *MYB* functions in flavonoid biosynthesis in other plants (Liu et al., 2015; Naik et al., 2022), although direct regulatory relationships require experimental confirmation.

### 3.7 Validation of differentially expressed genes through RT-qPCR

**Figure 10:**
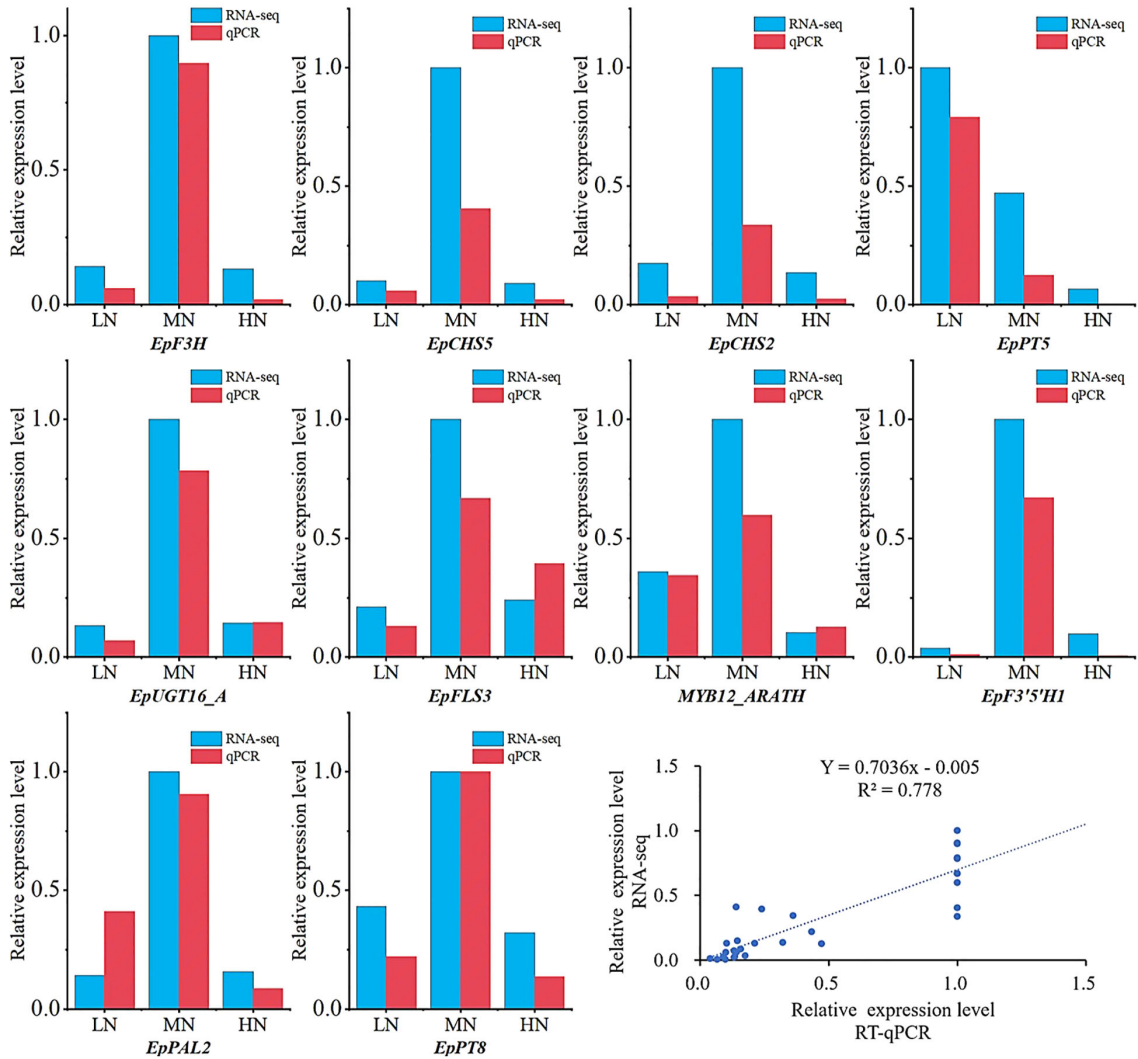
Validation of transcriptomic data by RT-qPCR at the S3 stage. Expression levels of selected genes: Transcriptomic data (blue bars, log_2_-transformed FPKM) and RT-qPCR results (red bars, normalized to reference genes). Correlation scatterplot between RT-qPCR (x-axis) and RNA-seq (y-axis) data.

To verify the reliability of the RNA-Seq data results, RT-qPCR was utilized to further examine the expression levels of differentially expressed genes (DEGs) between different treatments. Key genes primarily involved in flavonoid synthesis among the DEGs were selected for validation. The RT-qPCR results showed that the expression trends of the selected 10 DEGs were consistent with the transcriptome data, thereby confirming the reliability of the RNA-Seq data used in this study (**Figure 10**).

## 4 Discussion

Nitrogen is a crucial nutrient element for plant growth and productivity, but both excess and deficiency of nitrogen fertilizer can adversely affect plants. The medicinal plant *Epimedium pubescens* (*E. pubescens*), which has high medicinal value and is widely used in traditional Chinese medicine, primarily contains flavonoids as its active ingredients. However, the physiological and molecular mechanisms underlying the adaptation of *E. pubescens* to different nitrogen levels remain unclear. This study analyzed the effects of various nitrogen levels on the growth, carbon metabolites, and flavonoid accumulation in *E. pubescens*, and revealed two response mechanisms of the plant to nitrogen deficiency and excess stress.

### 4.1 Effects of nitrogen deficiency and excess nitrogen stress on the appearance and growth of *E. pubescens*

Under appropriate nitrogen levels (MN treatment, 7.5 mM NO_3_^-^), the growth and development of *E. pubescens* plants were normal, with healthy leaf color. However, under nitrogen deficiency stress, the leaves exhibited a pale green color, while under excessive nitrogen stress, the leaves turned scorched brown (**Figure 1A**). This change in appearance closely resembles the visual observations reported in pear seedlings grown hydroponically under varying nitrogen levels (Chen et al., 2018). The pale green color of leaves due to nitrogen deficiency stress is related to a decrease in chlorophyll content (**Figure 1E**). On the one hand, the nitrogen content in leaves significantly decreased (**Figure 2A**), resulting in a reduction of substances available for chlorophyll synthesis (Hudson et al., 2011); on the other hand, the “Porphyrin and chlorophyll metabolism” pathway in leaves was downregulated (**Supplementary Table S6**), leading to enhanced chlorophyll decomposition and conversion into mobile forms, which were then transported to new growth sites (Hudson et al., 2011; Havé et al., 2017).The browning of leaves caused by excessive nitrogen stress may be associated with water stress under HN treatment (Wang et al., 2021) and reduced potassium (K) and phosphorus (P) content (Chen et al., 2018), which leads to excessive ROS accumulation in leaves and subsequent cellular structural damage. However, in this study, the chlorophyll content of HN-treated leaves was higher than that of LN-treated leaves (**Figure 1E**), while both HN and LN treatments exhibited a decline in photosynthetic rate with no significant difference between them (**Figure 1D**). This discrepancy may be attributed to the multifaceted regulation of photosynthetic rate. Under LN conditions, the decline in photosynthetic rate is primarily due to chlorophyll limitation, whereas under HN conditions, factors such as water stress or excessive ROS accumulation may lead to cellular structural damage (Wang et al., 2021; Xing et al., 2021), or reduced Rubisco enzyme activity (Zhang et al., 2020). Further studies could investigate ultrastructural differences in leaves and changes in Rubisco enzyme activity to elucidate the underlying mechanisms of this phenomenon. Furthermore, under these two stresses, the downregulation of gene expression in the “Photosynthesis-antenna proteins” pathway in *E. pubescens* leaves leads to reduced efficiency in light energy harvesting and absorption, as well as impaired photoprotection ability (Bag, 2021), ultimately resulting in a decline in photosynthetic rate.

Nitrogen deficiency also increased the root-to-shoot ratio of the plants. Studies on soybeans and corn have confirmed that reduced nitrogen supply usually increases the accumulation of indole-3-acetic acid (IAA) in plant roots, thereby promoting root elongation (Caba et al., 2000; Tian et al., 2008). Additionally, low nitrogen stress may promote the transport of carbon assimilates and nitrogen nutrients to the roots (Lemoine et al., 2013; Liu et al., 2021b), which is beneficial for enhancing the foraging ability of plant roots (Giehl and von Wiren, 2014). Excessive nitrogen stress, on the other hand, results in a decrease in root biomass (**Figure 1C**), which may be related to a decrease in photosynthetic rate and carbon assimilation, as well as the plant’s reduction of nitrogen absorption by slowing down root growth (Cun et al., 2023). The effects of nitrogen deficiency and excessive nitrogen stress on plant roots are similar to those observed in studies on *Panax notoginseng* and *Cotton* (Chen et al., 2020; Cun et al., 2023).

### 4.2 Transcriptional strategies of *E. pubescens* in response to nitrogen deficiency and excess nitrogen stress

Under nitrogen deficiency and excess nitrogen stress, the number of differentially expressed genes (DEGs) obtained from *E. pubescens* leaves at S1-S3 stages (24 -48 DAT) exhibited different trends (**Figure 3**). Specifically, during the S2 stage (36 DAT), the number of DEGs under nitrogen deficiency stress (LN, 0 mM NO_3_^-^) peaked and then significantly decreased during the S3 stage (48 DAT), with a notable increase in the proportion of downregulated genes. In contrast, the number of DEGs under excess nitrogen treatment (HN, 22.5 mM NO_3_^-^) gradually increased over time, reaching a maximum at the S3 stage, with most DEGs showing upregulation (**Figure 3**). Therefore, it is reasonable to hypothesize that the transcriptional response strategies of *E. pubescens* leaves under nitrogen deficiency and excess nitrogen stress differ: under nitrogen deficiency stress, the leaves may reach their highest response level at the S2 stage (36 DAT), while under nitrogen excess stress, the most active response occurs at the S3 stage.

In the later stages of nitrogen deficiency stress (S3 stage, 48 DAT), the leaves transition from a storage reservoir of nutrients to a source of nutrient transport, with enhanced decomposition and senescence, and are discarded to decompose and produce nitrogen sources for other organs (Havé et al., 2017). Therefore, their transcriptional response weakens in the later stages (**Figure 3**). This is similar to the transcriptional response strategy found in previous studies of *E. pubescens* under phosphorus deficiency stress (Liu et al., 2024). The downregulation of cell wall-related gene expression and the “Porphyrin and chlorophyll metabolism” pathway in leaves under nitrogen deficiency stress further support this view (**Figure 4A**; **Supplementary Table S6**). Under excess nitrogen stress, plants may transfer excess nitrogen to senescent or mature leaves through nitrate transporter proteins and other pathways (**Supplementary Figure S2**) to alleviate stress pressure on other parts (such as bud). In the later stages of excess nitrogen stress (S3 stage, 48 DAT), the accumulation of excess nitrogen further increases, affecting more leaves and stimulating a more active transcriptional response.

The significant upregulation of the “Starch and sucrose metabolism” pathway at different stages under different nitrogen stresses also supports this view. “Starch and sucrose metabolism” is a common upregulated pathway in plants under stress responses, contributing to the production of more energy and soluble sugars to cope with stress (Li and Li, 2005; Chen et al., 2023). Specifically, this pathway was most significantly upregulated during the S2 stage (36 DAT) under nitrogen deficiency stress and during the S3 stage (48 DAT) under excess nitrogen stress (**Figures 4B, D**). Consequently, the content of carbon metabolites (soluble sugars and starch) decreased in leaves under nitrogen deficiency and nitrogen excess stress (**Figure 2A**).

Both nitrogen deficiency and excess stress led to the downregulation of the “Flavonoid biosynthesis” pathway in the later stages (**Figures 4B, D**, **7B**), which accounted for the decrease in Icariin-type flavonoids content in the leaves (**Figure 2B**). The upregulation of “Anthocyanin biosynthesis” pathway under nitrogen deficiency stress, which may be related to the damage of the leaf photosynthetic system and the decrease in chlorophyll content, could protect the leaves from light damage (Ferreira Da Silva et al., 2012). In the later stages of excess nitrogen stress, the “Phenylpropanoid biosynthesis” and “Cutin, suberine, and wax biosynthesis” pathways were upregulated in *E. pubescens* leaves (**Figures 4D**, **5**). This upregulation might be related to the water stress experienced by the leaves (Wang et al., 2021). It promoted lignin synthesis, enhanced the leatheriness and waxiness of the leaves, which helped reduce water loss and increase the plant’s tolerance to stress (Wu et al., 2022).

Different nitrogen levels affected the ion binding of nutrient elements within plants. In this study, “copper ion binding” was upregulated under nitrogen deficiency stress, while “calcium ion binding” and “iron ion binding” were upregulated under excess nitrogen stress (**Figure 4C**). Studies on pear cultivation at different nitrogen levels found significant changes in the content of calcium, magnesium, and iron ions in leaves (Chen et al., 2018). This might be related to the synergistic absorption and antagonism between nitrogen and other mineral elements (Feil and B Nziger, 1993).

Furthermore, the “Circadian rhythm-plant” pathway was downregulated in the later stages of excess nitrogen stress. Nitrate serves as a non-photonic signal in the plant’s circadian rhythm system (Roenneberg and Rehman, 1996), and similar phenomena have been observed in other plants. Cultivation of walnut seedlings at different nitrogen levels found that nitrogen signals might affect the circadian rhythm by regulating genes such as CRY1 and LHY (Song et al., 2022).

### 4.3 WGCNA reveals common modules in *E. pubescens* responses to nitrogen deficiency and excess stress

WGCNA revealed gene modules (grey60 module) with similar expression patterns under nitrogen deficiency and excess stress (**Figure 6C**). The KEGG enrichment results of the grey60 module demonstrated that, in addition to pathways related to carbon and nitrogen metabolism (such as amino acid synthesis and carbohydrate metabolism), “Biosynthesis of cofactors” was also enriched. This indicated that both nitrogen stresses affected carbon and nitrogen metabolism in *E. pubescens* leaves, and further, due to the impact on cofactor biosynthesis, they potentially influenced enzyme activities in other metabolic processes. Additionally, the enrichment of “Porphyrin metabolism” suggested that, both nitrogen deficiency and excess stresses impact chlorophyll structure. “Sphingolipid metabolism” was also enriched (**Figure 6D**), suggesting that both stresses might disrupt the structure of cell membranes. While nitrogen deficiency might lead to chlorophyll degradation for reuse, excess nitrogen stress could damage chloroplast structure due to ROS accumulation (**Figure 6D**). Meanwhile, in the later stages of excess nitrogen stress, the upregulation of the “Glutathione metabolism” pathway (**Figure 4C**) helps alleviate ROS accumulation (Gao et al., 2023), further demonstrating the complex interplay between nitrogen status and metabolic pathways in *E. pubescens*.

In addition to generating active transcriptional responses, *E. pubescens* leaves under these two stresses also responded through post-transcriptional regulation, as evidenced by the enrichment of “Aminoacyl-tRNA biosynthesis” and “RNA degradation” (**Figure 6D**).

### 4.4 Nitrogen deficiency and excess nitrogen stress significantly affect the carbon-nitrogen metabolism balance and icariin-type flavonoid metabolism in *E. pubescens*

Under nitrogen deficiency and excess nitrogen stress, significant changes occur in carbon-nitrogen metabolism. Photosynthesis, the initial pathway of plant carbon metabolism, is reduced in leaves under both nitrogen deficiency and excess stress (**Figure 1D**). As the main products of photosynthesis and important participants in carbon metabolism processes, starch and soluble sugars exhibit the same trend as photosynthesis under different nitrogen levels. Compared to optimal nitrogen levels, the contents of starch and soluble sugars are significantly reduced under nitrogen deficiency and excess stress (**Figure 2A**). This change is related to both the impairment of photosynthesis in plants and the regulation of internal carbon metabolism-related enzyme activity and gene expression by nitrogen levels (Zhang et al., 2020). Transcriptome analysis results show that “Starch and Sucrose Metabolism” is the primary carbon metabolism pathway affected in *E. pubescens* under nitrogen deficiency and excess stress (**Figures 4B, D**). After optimal nitrogen application, the nitrogen content in leaves significantly increases, but excess nitrogen application has a limited effect on further increasing leaf nitrogen content (**Figure 2A**), resulting in decreased nitrogen use efficiency in plants. This is because plants have limited nitrogen absorption rates and capacities, and the remaining excess nitrogen after absorption is lost, leading to reduced nitrogen use efficiency. In summary, optimal nitrogen application promotes the accumulation of carbon metabolites in plants, while nitrogen deficiency and excess stress inhibit photosynthesis in *E. pubescens*, disrupting its internal carbon-nitrogen metabolism balance and reducing plant nitrogen use efficiency.

The disruption of carbon-nitrogen metabolic balance has impacts on various aspects such as the growth and development of *E. pubescens* and flavonoid metabolism. The metabolic pathway diagram (**Figure 7**) illustrates that carbon and nitrogen metabolism provide substrates for flavonoid synthesis. In particular, the accumulation of carbon metabolites plays a crucial role in providing the carbon skeleton. The effects of nitrogen deficiency and excessive nitrogen stress on the carbon-nitrogen metabolic balance result in a reduction in carbon metabolites, significantly decreasing the carbon substrate source for the flavonoid synthesis pathway, thereby hindering the synthesis and glycosylation modification of Icariin-type flavonoids and leading to a decrease in its content (**Figure 7B**). Similar results were also observed in studies of *Cyclocarya paliurus* (*C. paliurus*) and *Coreopsis tinctoria* (*C. tinctoria*). Deng B (Deng et al., 2019) found that the flavonoid content in *C. paliurus* was significantly positively correlated with total carbon content and starch levels. At moderate nitrogen levels, both starch and flavonoid contents in *C. paliurus* reached their highest levels. As nitrogen levels continued to increase, more carbon flowed into nitrogen metabolism, reducing carbon substrates and subsequently decreasing flavonoid content. Li Z (Li et al., 2021) found that low to moderate nitrogen levels promoted the accumulation of carbohydrates in *C. tinctoria* but reduced the content of nitrogen metabolites. Flavonoid content was significantly positively correlated with carbohydrate content and negatively correlated with nitrogen metabolite content. This confirms that insufficient or excessive nitrogen levels inhibit the accumulation of carbon-containing metabolites. The accumulation of carbon-containing compounds is a necessary but not sufficient condition for the increase of secondary metabolism, which aligns with the Growth-Differentiation Balance Hypothesis (Glynn et al., 2007), which suggests that appropriate nutrient levels (nitrogen levels) promote a balance between growth (primary metabolism) and differentiation (secondary metabolism). Transcriptional results showed that both LN and HN treatments significantly downregulated the expression of genes related to the flavonoid synthesis pathway at 48d, with most of the key genes in the Icariin-type flavonoid synthesis pathway showing downregulation (**Figure 7B**). RT-qPCR validated these results (**Figure 10**).

The downregulation of gene expression related to the flavonoid pathway under excessive nitrogen stress is consistent with many research findings, such as Xu X (Xu et al., 2022) found that “Fuji” Apple under high nitrogen downregulated the expression of structural genes (e.g., *MdPALs*, *Md4CLs*, *MdF3H*) and their regulators (*MdMYBs* and *MdbHLHs*) involved in the anthocyanin synthesis pathway. Liu J (Liu et al., 2021a) found that under high nitrogen levels, the expression levels of genes related to flavonoid synthesis (*PAL*, *CHI*, *CHS*, *F3H*) in hydroponic tea plant branches significantly declined. However, the downregulation of gene expression related to the flavonoid synthesis pathway in the leaves of *E. pubescens* caused by nitrogen deficiency is contrary to the results observed in many other species. For example, Shao CH (Shao et al., 2020) found that under nitrogen deficiency, the expression levels of key genes involved in the flavonoid synthesis pathway, such as *DFR*, *CHS*, and *CHFI*, were significantly upregulated in *Rice*. Huang H (Huang et al., 2018) found that nitrogen deficiency caused tea plants to accumulate various flavonoids and increased the expression levels of hub genes involved in flavonoid synthesis, such as *F3H*, *FNS*, *UFGT*, *bHLH35*, and *bHLH36*. This indicates that different species have different response mechanisms to nitrogen deficiency stress, and the trends in the expression changes of flavonoid synthesis-related genes are not conserved in the response to nitrogen deficiency stress among species.

### 4.5 Integrated regulatory network of carbon-nitrogen metabolism and icariin-type flavonoid biosynthesis

By constructing correlation networks of genes related to carbon and nitrogen metabolism, as well as flavonoid metabolism, from the LN and HN treatments (nitrogen deficiency and excessive nitrogen stress) during the S3 stage (48d) (**Figures 7**, **8**), we found that the hub genes regulating carbon metabolism in response to nitrogen levels are mainly involved in starch degradation and phosphorylation and decomposition of glucose, fructose, etc., such as Ebr01G064020 (α-amylase) and Ebr01G044770 (fructose-1,6-bisphosphatase). This is consistent with the research on *C. paliurus* by Deng B (Deng et al., 2019) and the study on *C. tinctoria* by Li Z (Li et al., 2021), indicating that starch content is the primary carbon metabolite affected by different nitrogen levels. The hub genes of nitrogen metabolism are primarily associated with amino acid synthesis, such as Ebr04G062170 (amidase) and Ebr02G065060 (aromatic aminotransferase). This aligns with the findings by Liu J (Liu et al., 2021a) that increased nitrogen levels lead to an increase in the free content of various amino acids in hydroponic tea plant branches, suggesting that nitrogen levels mainly influence amino acid content during nitrogen metabolism. The hub genes involved in the synthesis of Icariin-type flavonoids glycosides are related to the conversion of chalcones into flavones and the synthetic modification of flavones, such as Ebr06G044290 (*UGT*, UDP-glycosyltransferase) and Ebr04G062950 (*EpF3H*, flavanone 3-hydroxylase). These findings are similar to those of most studies affecting flavonoid genes, with significant changes in the expression levels of genes such as *F3H*, which are involved in the conversion of chalcones into flavones in tea plants under different nitrogen levels (Huang et al., 2018; Liu et al., 2021b). Research on Salix fluviatilis indicates that nitrogen deposition promotes methylation of flavonoids in male plants and glycosylation in females (Cai et al., 2023).

Furthermore, our study revealed differential expression of *MYB1* and *MYB12* under varying nitrogen stress conditions, with significant correlations to genes involved in the icariin-type flavonoid biosynthesis pathway, including *UGT* (Ebr06G045440), *UGT* (Ebr06G044290), *EpF3H* (Ebr04G062950), and *EpCHS5* (Ebr03G073940). However, while these high correlations suggest potential regulatory interactions, they do not confirm direct regulation of these genes by *MYB1* or *MYB12*, which requires further functional validation. Notably, in *Crocosmia crocosmiiflora*, *CcMYB1* has been experimentally shown to enhance 4′-hydroxyflavonoid biosynthesis (Irmisch et al., 2019), and *MYB12* is known to activate the expression of *CHS*, *CHI*, *F3H*, and *FLS*, playing a critical role in UV-B stress responses (Mehrtens et al., 2005). Nevertheless, due to the lack of an established genetic transformation system in *E. pubescens*, the specific roles of *MYB1* and *MYB12* in nitrogen stress adaptation within this species remain to be further elucidated.

## 5 Conclusion

Through comparative physiological, biochemical, and transcriptomic analyses of *E. pubescens* under nitrogen deficiency, excess nitrogen stress, and moderate nitrogen conditions, this study revealed for the first time the multifaceted effects of extreme nitrogen levels on growth performance and secondary metabolite accumulation in *E. pubescens*. We elucidated the relationship between carbon/nitrogen metabolism and icariin-type flavonoid biosynthesis and identified key genes within their interconnected regulatory networks. Notably, critical candidate genes (e.g., *Ebr04G062950*: *EpF3H*; *Ebr06G044290*: *UGT*) were identified as pivotal regulators of nitrogen-mediated icariin-flavonoid biosynthesis in *E. pubescens* leaves. These findings provide a critical foundation for understanding the nitrogen-dependent regulatory mechanisms governing medicinal plant growth and secondary metabolism. Furthermore, this work offers theoretical guidance for optimizing nitrogen fertilization strategies and breeding nitrogen-efficient cultivars in *E. pubescens* production, with the identified genes serving as valuable genetic resources for future research.

## Data Availability

The datasets presented in this study can be found in online repositories. The names of the repository/repositories and accession number(s) can be found below: https://www.ncbi.nlm.nih.gov/, GSE273547.
