# Supplementary material for: Integrative transcriptomic-physiological analysis deciphers nitrogen-mediated carbon reallocation balancing growth and flavonoid metabolism in Epimedium pubescens
Source: Front Plant Sci. 2025 May 8;16:1539445. doi: 10.3389/fpls.2025.1539445 (PMC12095337; doi:10.3389/fpls.2025.1539445)
Supplement: Supplementary file 1 [file SupplementaryFile1.docx]

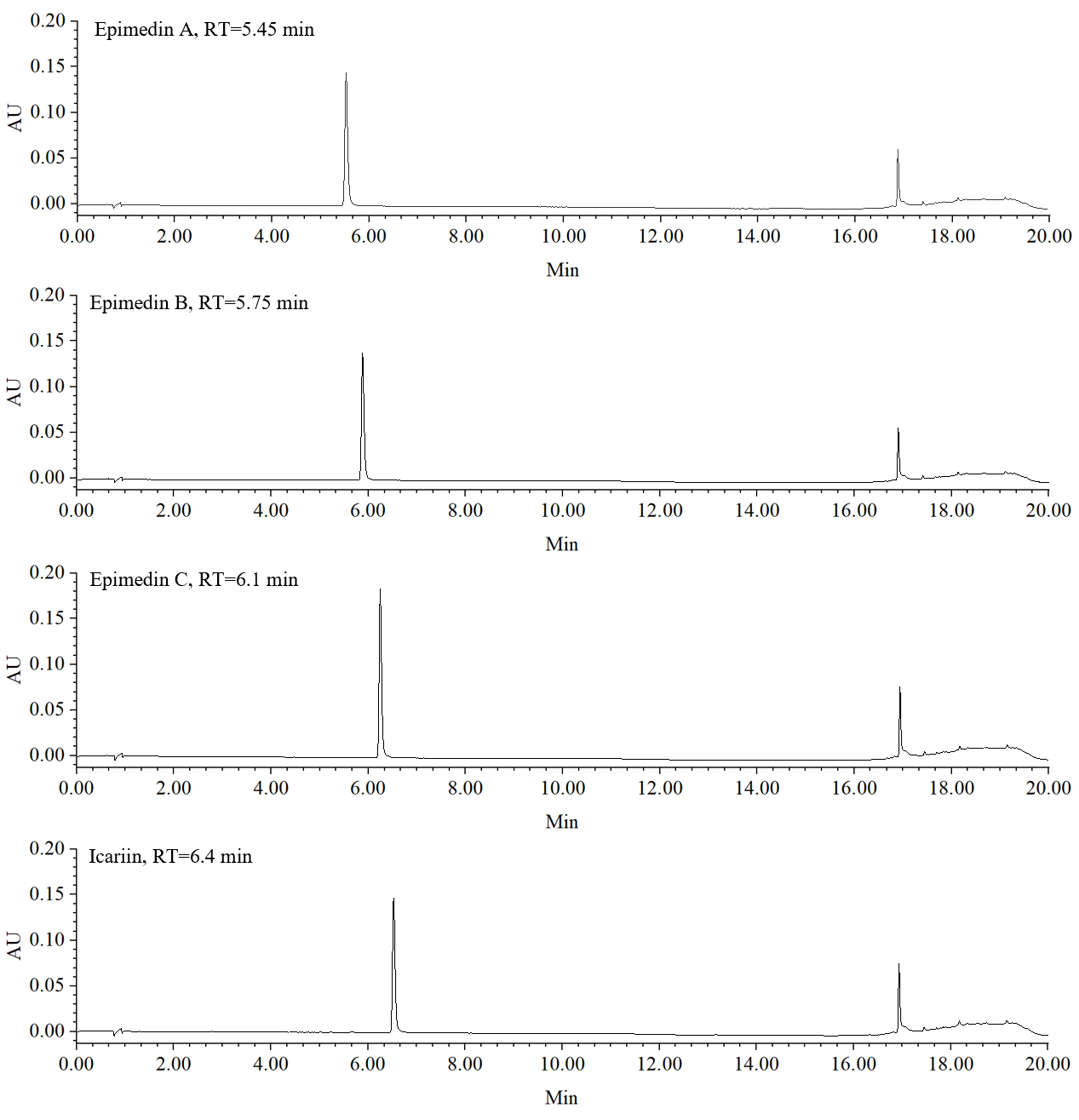


#### Fig.S1 Chromatograms and retention times of Epimedin A, Epimedin B, Epimedin C, and Icariin using Ultra-Performance Liquid Chromatography (UPLC).


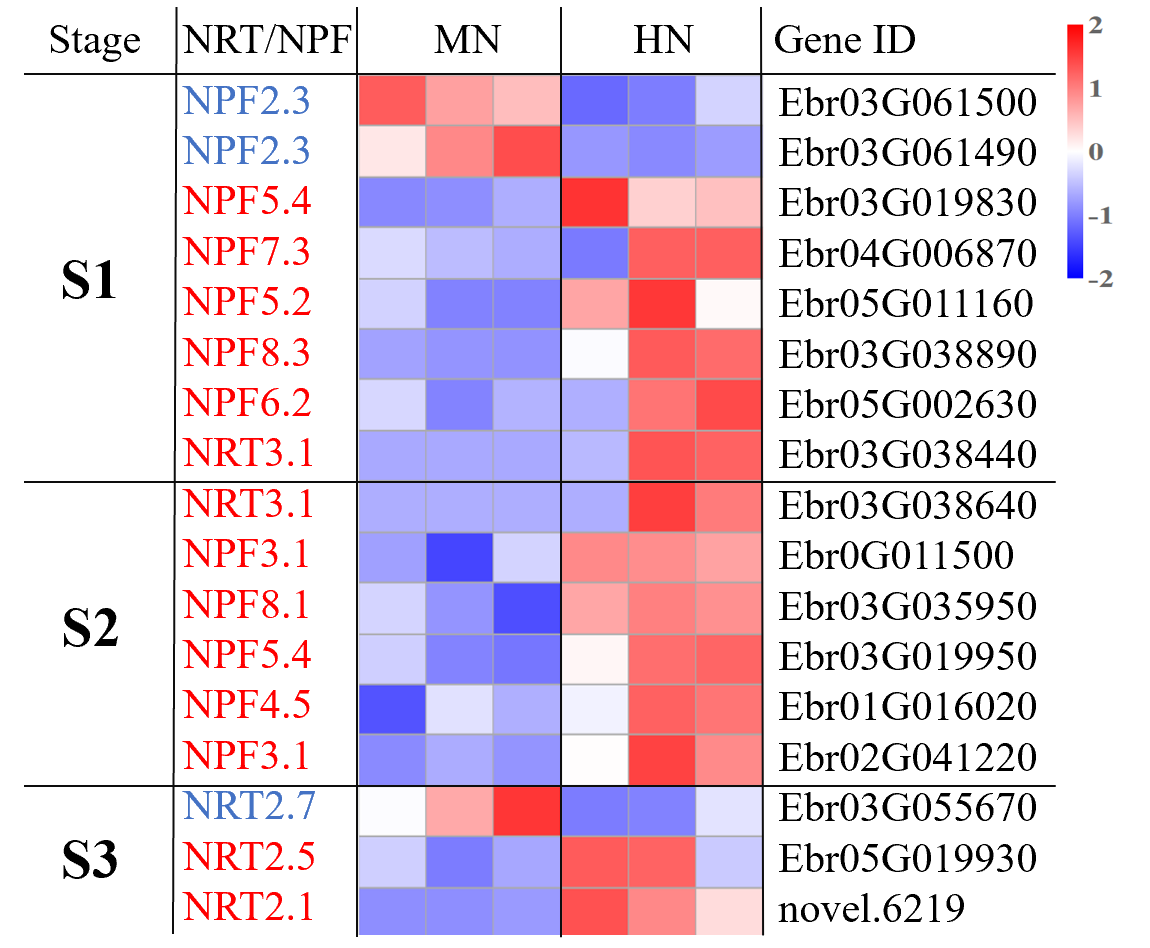


#### Fig.S2 Heatmap showing the expression changes of genes encoding nitrate transporter-related proteins in MN vs HN.
